# From Jekyll to Hyde and Beyond: Hydrogen’s Multifaceted Role in Passivation, H-Induced Breakdown, and Charging of Amorphous Silicon Nitride

**DOI:** 10.1021/acs.jpclett.3c03376

**Published:** 2024-01-18

**Authors:** Jonathon Cottom, Lukas Hückmann, Emilia Olsson, Jörg Meyer

**Affiliations:** † Leiden Institute of Chemistry, Gorlaeus Laboratories, Leiden University, P.O. Box 9502, 2300 RA Leiden, The Netherlands; ‡ Advanced Research Center for Nanolithography, Science Park 106, 1098 XG Amsterdam, The Netherlands; ¶ Institute for Theoretical Physics, University of Amsterdam, Postbus 94485, 1090 GL Amsterdam, The Netherlands

## Abstract

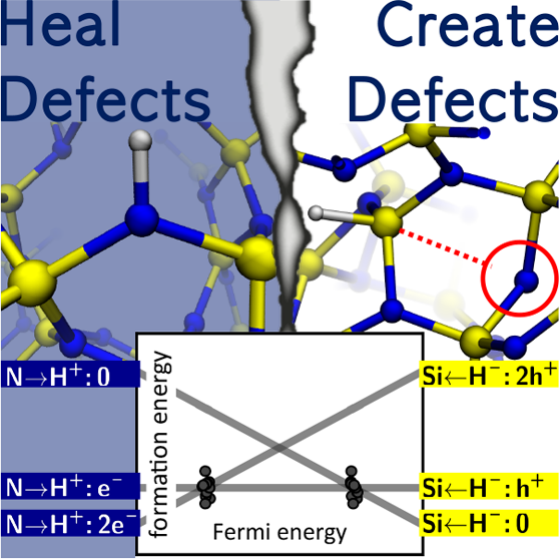

In semiconductor devices, hydrogen has traditionally been viewed as a panacea for defects, being adept at neutralizing dangling bonds and consequently purging the related states from the band gap. With amorphous silicon nitride (a-Si_3_N_4_)—a material critical for electronic, optical, and mechanical applications—this belief holds true as hydrogen passivates both silicon and nitrogen dangling bonds. However, there is more to the story. Our density functional theory calculations unveil hydrogen’s multifaceted role upon incorporation in a-Si_3_N_4_. On the “Jekyll” side, hydrogen atoms are indeed restorative, healing coordination defects in a-Si_3_N_4_. However, “Hyde” emerges as hydrogen induces Si–N bond breaking, particularly in strained regions of the amorphous network. Beyond these dual roles, our study reveals an intricate balance between hydrogen defect centers and intrinsic charge traps that already exist in pristine a-Si_3_N_4_: the excess charges provided by the H atoms result in charging of the a-Si_3_N_4_ dielectric layer.

Silicon nitride has been a technologically important material for a number of years due in no small part to its attractive chemical, mechanical, and electronic properties. This has led to it being deployed in a broad range of applications covering wear-resistant coatings,^1^ electronic devices (MOSFETs and MEMs),^2,3^ high-energy optics,^4−6^ integrated photonics,^7,8^ and lithography.^9−11^ For a small number of applications, silicon nitride is used in the crystalline phase.^12,13^ However, in most use cases it is deployed as an amorphous thin film (a-Si_3_N_4_).^3,14−19^ The addition of hydrogen can be intentional, as is the case with photovoltaics and electronic devices that undergo a H-anneal step. This passivation step has been shown to be both a blessing, passivating dangling bonds,^20−22^ and a curse, affecting structural stability and altering the charge trapping properties.^23−26^ Alternatively, H can be incorporated unintentionally as a result of exposure to the environment, such as is the case in lithography.^4−6,9−11,27^ Finally, a certain concentration of H will be incorporated from the precursors during thin film growth by chemical vapor deposition, typically SiH_4_ and its derivatives. Regardless of how H is introduced to a-Si_3_N_4_, at the atomic scale an understanding of H incorporation and the concomitant modification of the material’s properties has not been established.

Notwithstanding, experimental and theoretical studies have provided a number of important insights. H defects have been linked to the passivation of dangling bonds within the a-Si_3_N_4_ network,^20−22,28−38^ based on a reduction of the observed concentration of paramagnetic centers upon increases in Si–H vs N–H concentration.^39,40^ This reduction in undercoordinated Si and N has additionally been linked to the observed increase in breakdown strength and perhaps surprisingly to a decrease in the a-Si_3_N_4_ film’s tensile stress.^23^ In addition, the post-deposition treatment with H leads to significant changes in the observed photoluminescence and EPR spectra.^26,41,42^ The latter results suggest that H plays a dual role, leading to a reduction in midgap recombination centers while favoring or introducing others, resulting in both radiative and nonradiative recombination pathways.^26^ Conversely, it has been observed that as the H concentration in the a-Si_3_N_4_ film increases, the temperature stability of the film decreases.^24,25,43,44^ The disparate nature of H incorporation with respect to the properties of a-Si_3_N_4_ suggests a range of atomic environments to be of relevance, beyond just the passivation of dangling bonds.

H incorporation in a variety of wide band gap oxides has been extensively studied.^45−54^ Therefore, it is tempting to look for parallels between nitrides and oxides. In particular, the interaction of H with SiO_2_ shows a number of similar trends as for a-Si_3_N_4_ summarized above, reducing the number of coordination defects while forming new defect centers.^52−55^ The formation of new defect centers and the disruption of the silica lattice are suggested to result in the observed hydrolytic weakening, whereby exposure to H_2_ and H_2_O induces a significant weakening in SiO_2_ and a-Si_3_N_4_.^43,44,56,57^ Theoretical studies have provided atomistic insights into H incorporation in SiO_2_ and identified a mechanism for the H-induced bond breaking.^52−55^ In general, for a broad range of oxides, H defects fit into two main categories. The first category is amphoteric defects, which result in H states deep in the band gap that typically exhibit negative *U* character, whereby the charge state (+/−) is dictated by the Fermi energy in the device. Alternatively, H can act as an n-type dopant introducing states in the vicinity of the conduction band minimum (CBM). A link between the (+/−) H charge transition level (CTL) and a universal value has been postulated, which further is related to the charge neutrality level (3.0,^49^ 3.9,^51^ and 4.5^48^ eV below the vacuum level, depending on the electronic structure approach employed). Li and Robertson^51^ noted this description and the concomitant universal value breakdown when the H is able to form a dative bond with the O-site. Considering the fact that chemical bonding in a-Si_3_N_4_ is markedly different compared to that in a-SiO_2_, it is an open question of whether (and if so how) the aforementioned prevalent description of H defects can be extended to this class of materials.

Here we present a systematic study of H incorporation in a-Si_3_N_4_ as a function of the local atomic environment. To ensure that a statistically meaningful range of H incorporation sites are considered, we build on the sampling scheme developed in our previous work.^58^ We study the stability, structural features, charging behavior, and interplay between the extrinsic H defects and intrinsic charge trapping as characterized before.^58^ This allows us to quantify the multifaceted role H plays in a-Si_3_N_4_, including passivation and the hitherto unknown structural modifications and H-induced breakdown of the network. Our analysis underscores a pronounced interplay between H and existing charge traps within the amorphous network. Contrary to the role of H as either an amphoteric defect center or an n-type dopant, more typically encountered, this nuanced behavior finds its roots in semilocalized states proximate to the CBM and VBM, resulting in an electrically inactive H center. Upon the incorporation of charge, predominantly donated by hydrogen, the excess charge is then localized at intrinsic traps.

*H Incorporation in β-Si_3_N_4_*. As a starting point, we briefly revisit hydrogen incorporation in the most stable crystalline phase (β-Si_3_N_4_), which has been previously studied by Di Valentin et al.^33^ and Grillo et al.^59^ To facilitate the comparison with a-Si_3_N_4_, we have performed calculations with our computational setup. Full details are included in the Supporting Information, and a brief summary is given here: In β-Si_3_N_4_, hydrogen forms a negative-*U* (*U* ≈ −0.5 eV) amphoteric defect familiar from a broad range of oxides,^48,51^ most notably SiO_2_. Depending upon the Fermi energy, either the H^+^ or H^–^ is favored. At *E*_F_ < 3.28 eV, the H^+^ is favored, sitting at a N site with an N–H separation of 1.05 Å. Above 3.28 eV, the H^–^ is favored, sitting adjacent to a Si with a separation of 1.5 Å. Finally, for the neutral charge, while never energetically favored, the H adopts an interstitial configuration This maximizes the distance to the neighboring ions, resulting in a Si/N–H separation of 2.4 Å. The (+/−) CTL sits approximately 4.15 eV below the vacuum level and is in reasonable agreement with the observations of van de Walle and Neugebauer^48^ as well as Li and Robertson.^51^ Interestingly and in contrast to the crystalline system, the noninteracting H^0^ interstitial does not form in a-Si_3_N_4_. Instead, both N- and Si-centered H defects are found and discussed below, starting from the neutral charge state in both cases.

*N–H Defects*. Two main types of N–H defects are dictated by the original coordination of the reference site in the precursor geometry (Figure 1a,b):

1. H incorporation on two-coordinate N resulting in a passivation-type interaction (Si_2_N–H_pass_), and
2. H incorporation on three-coordinate N resulting in a tetrahedral-like distortion of the planar N forming a hydrogen interstitial (Si_3_N–H_i–N_).

**Figure 1:**
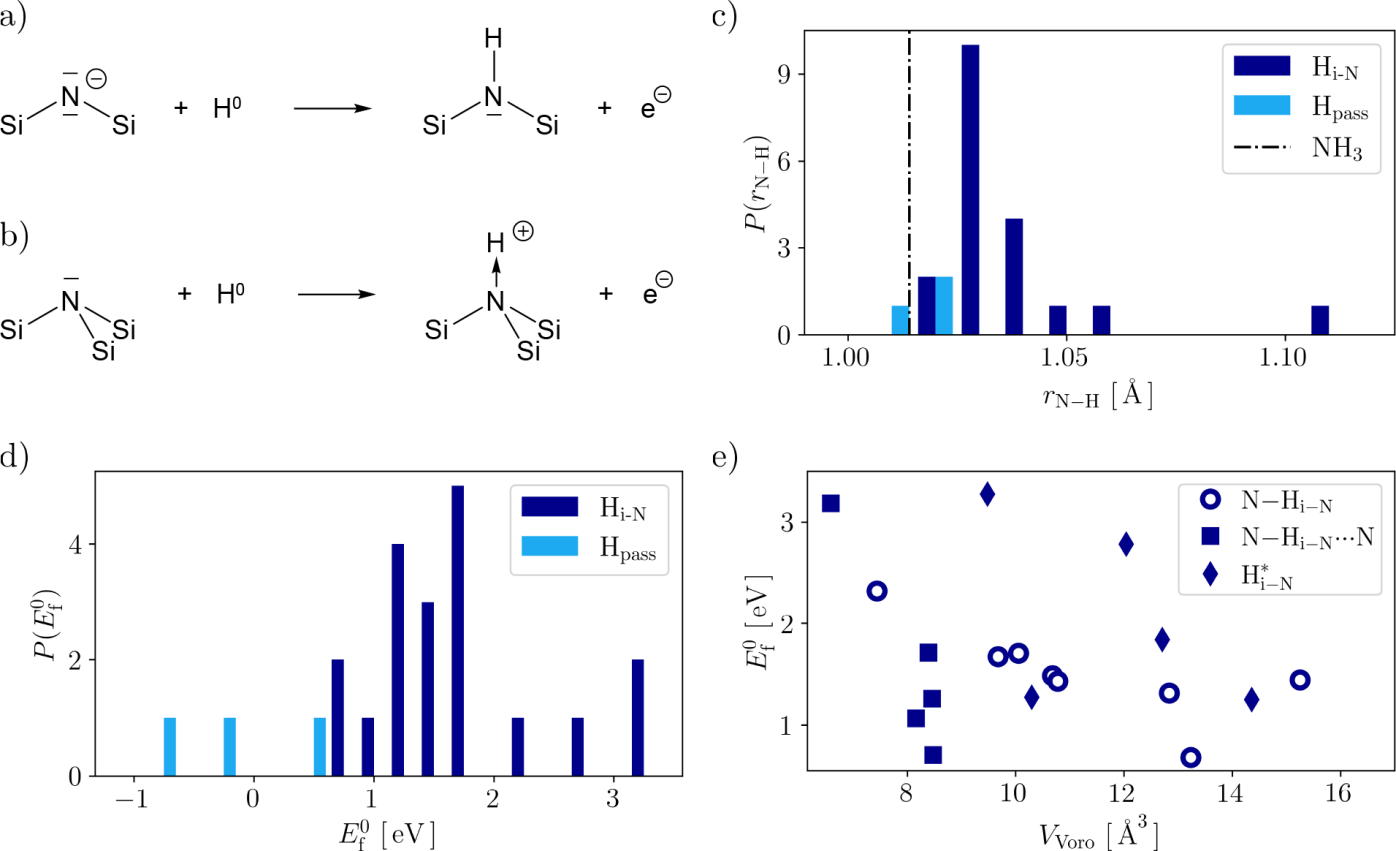
Schematic representation of N–H defect configurations on two- and three-coordinate host atoms forming a) H passivation sites (H_pass_) and b) H interstitial sites (H_i-N_) in the neutral charge state (*q* = 0). The excess electron of the neutral hydrogen atom (H^0^) is detached from the site and trapped at an intrinsic defect in the material. c) Distribution *P* of the N–H bond lengths for H_pass_ (light blue) and H_i-N_ (dark blue). For comparison, the N–H bond length of ammonia (NH_3_) is indicated as the dashed–dotted line.^60^ d) Distribution *P* of the formation energies *E*_f_^0^ (same color code as in c). e) *E*_f_^0^ as a function of the Voronoi volume *V*_Voro_ for H_i-N_. This group of defects is further decomposed into different subgroups: Interstitials with a hydrogen bridge-like site N–H_i–N_···N, which are marked as squares (28%). Those inducing new electron trap sites not present in the pristine cell (H _iN_^*^) are marked as diamonds (23%). The remainder (49%), where H interacts with a single N and the electron is trapped at the original H-free trap site, are labeled by N–H_i–N_ and marked as empty circles.

The N–H bond lengths for both cases sit in a tight range between 1.01 and 1.06 Å with one outlier at 1.10 Å and the mean at 1.03 Å (Figure 1c). This makes the bonds marginally elongated compared to the N–H bond length in ammonia.^60^ As to be expected, the very few H_pass_ defects present in our cell form the closest match and also come with the most favorable formation energies as shown in Figure 1d. Again for both H_pass_ and H_i-N_, incorporation coincides with negligible structural relaxation as the adjacent N–Si bonds lengthen only by 0.11 Å (∼6%) on average. In contrast, the formation energies for H_i-N_ extend over a considerable range of 0.68 to 3.27 eV (see Figure 1d). The fact that the H_i-N_ sites are uniform in terms of the N–H bond length and coordination geometry of the N-host indicates that the local environment of the host site dictates *E*_f_^0^. This is confirmed in Figure 1e, illustrating that *E*_f_^0^ scales with the local steric environment, described by the Voronoi volume (*V*_Voro_) of the incorporation site. In general (empty circles), a high degree of steric crowding (small *V*_Voro_) results in high *E*_f_^0^, and a small degree of steric crowding (large *V*_Voro_) results in low *E*_f_^0^. Sites with a nitrogen atom opposite to the host atom lead to a bridge-like interaction (N–H···N). The H···N distance is between 1.68 and 1.85 Å with an N–H···N angle between 1.3 and 32.6°. These structural arrangements allow H incorporation in sterically crowded regions of the lattice with a reduced energetic penalty (filled squares in Figure 1e). Finally, a subset of H_i-N_ defects can be distinguished, creating new states for electron trapping that are not present in the pristine cell (*vide infra*). As illustrated by the filled diamonds in Figure 1e, this subset follows the aforementioned trend less clearly.

Consideration of the +1 and −1 charge states shows that the H geometry is decoupled from the charge state with no change in either the N–H bond length or orientation. The reason for this becomes apparent by considering the electronic density of states (DOS) in the neutral charge state as shown in Figure 2a for a representative configuration. The H-induced electronic states are smeared out, with no significant contributions to the DOS below the VBM and empty states ∼1.0 eV above the CBM. As there are no H states within the band gap, H–N is electronically inactive, and the charge state of H is thus independent of the device Fermi level, unlike for amphoteric H defects. Instead, here H is datively bound to N, resulting in a H^+^. The excess electron provided by the hydrogen is localized at an intrinsic trap site previously identified and characterized (strained SiN_4_ tetrahedra, see Figure 2b).^58^ For the majority of cases (77%, given by N–H_i–N_ and N–H_i–N_···N in Figure 1e), further examination of the spin density, Mulliken charge, and DOS in the same way as described in detail in our previous work^58^ reveals an unpaired electron localizing consistently on the same intrinsic trap site in our simulation cell as illustrated in Figure 2b and Figure S3 in the Supporting Information. The fact that charge trapping is thus dominated by a feature of pristine a-Si_3_N_4_ rationalizes the lack of geometric relaxation of H defects upon charging and the extremely small spread of the CTLs shown in Figure 2c. Both are distinct features of H incorporation enabled by dative bonding and are clearly at odds with amphoteric H defects. In the remaining cases (23% H_iN_^*^ in Figure 1e, not considering H_pass_), the structural distortion caused by the H incorporation induces an alternative precursor site, centered on a single distorted Si tetrahedron where the electron localizes. In both cases, this intimate link between (effectively) adding or removing charges and structural relaxation is equivalent to intrinsic charge trapping in the H-free cell. It is important to note that in the −1 charge state both electrons occupy the trap state, forming a bipolaron. This was not included in our previous study because only single electron/hole trapping was considered in the pristine cell.^58^ The local structure of the trap site experiences pronounced relaxation, resulting in a breaking of the Si–N (Δ*r*_SiN_ = 0.55 Å), and the Si is back-projected in such a way that it interacts with an adjacent Si neighbor. Nevertheless, the Si–Si distance is 2.26 Å and the structure is similar to that of N_3_Si-SiN_*x*∈{3,4}_ previously described.^58^

**Figure 2:**
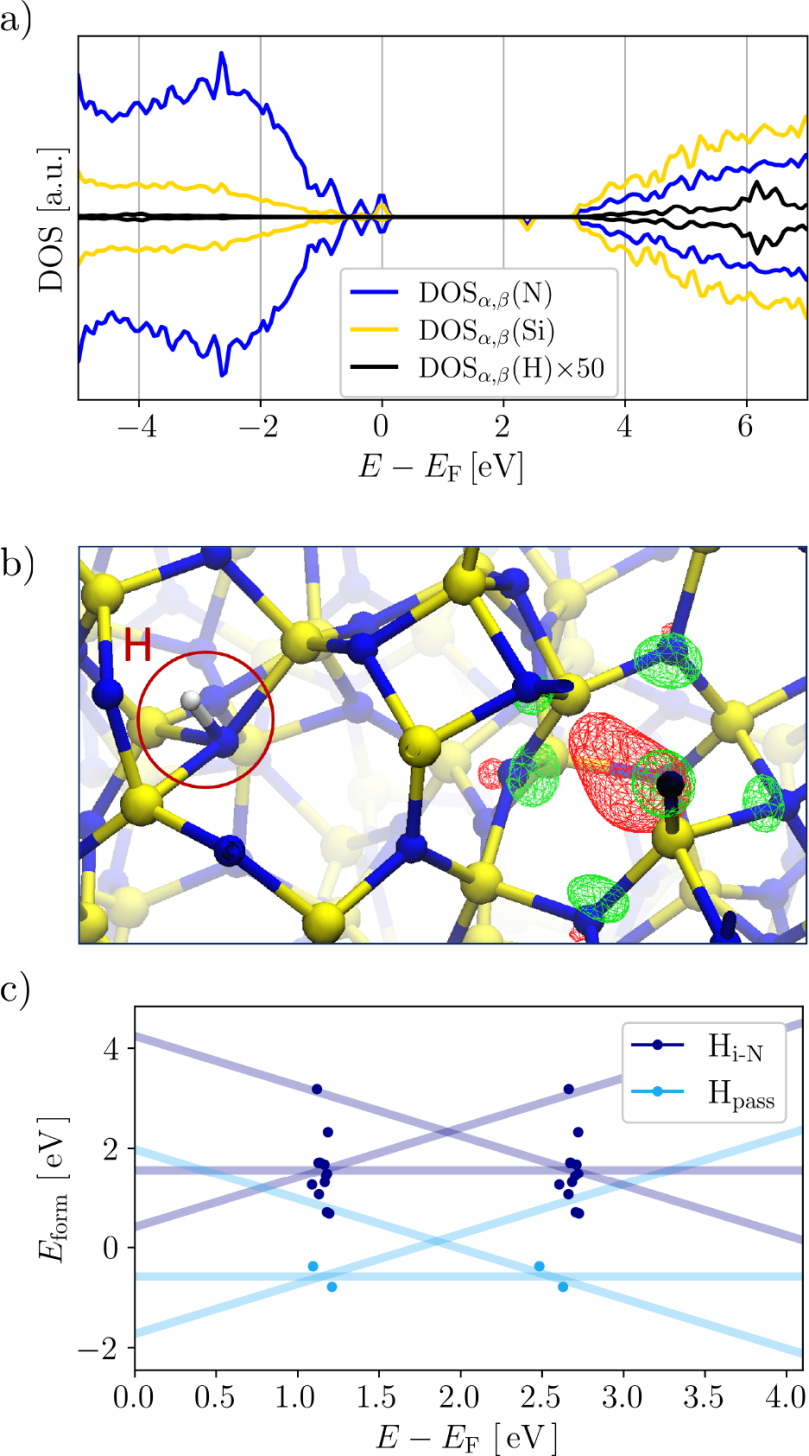
a) Projected electronic density of states (DOS) of Si (yellow), N (blue), and H (black) in a single representative neutral H_i-N_ configuration relative to the Fermi level *E*_F_. The DOS of H is amplified by a factor of 50 for visibility. b) Representative example of the localized state on a Si atom formed independently from the N–H site in the neutral charge state. Si is colored yellow, N blue, and H white. The respective spin channels are colored red and green. c) Formation energies *E*_form_ as a function of the Fermi level *E*_F_ for the H_pass_ and H_i-N_ defects in the +1, neutral, and −1 charge states (same color code as in Figure 1f). Solid lines mark the average over the entire ensemble, and points indicate the individual +1/0 (average 1.2 eV) and 0/–1 (average 2.6 eV) charge transition levels. Samples inducing new electron trap sites not present in the pristine cell (H _iN_^*^ in Figure 1e) are not included.

*Si–H Defects*. We have identified and schematically depicted three main classes of defects in Figure 3a–c:

1. H interacting with a single fully coordinated Si atom (N_4_Si–H_i-Si_),
2. H bridging two adjacent Si atoms, in addition to their regular link via a N atom in the a-Si_3_N_4_ network (N_4_Si–H_bri_–SiN_4_), and
3. H inserting across a strained Si–N bond, resulting in H–SiN_3_ and an adjacent two-coordinate N (N_3_Si–H_ins_).

**Figure 3:**
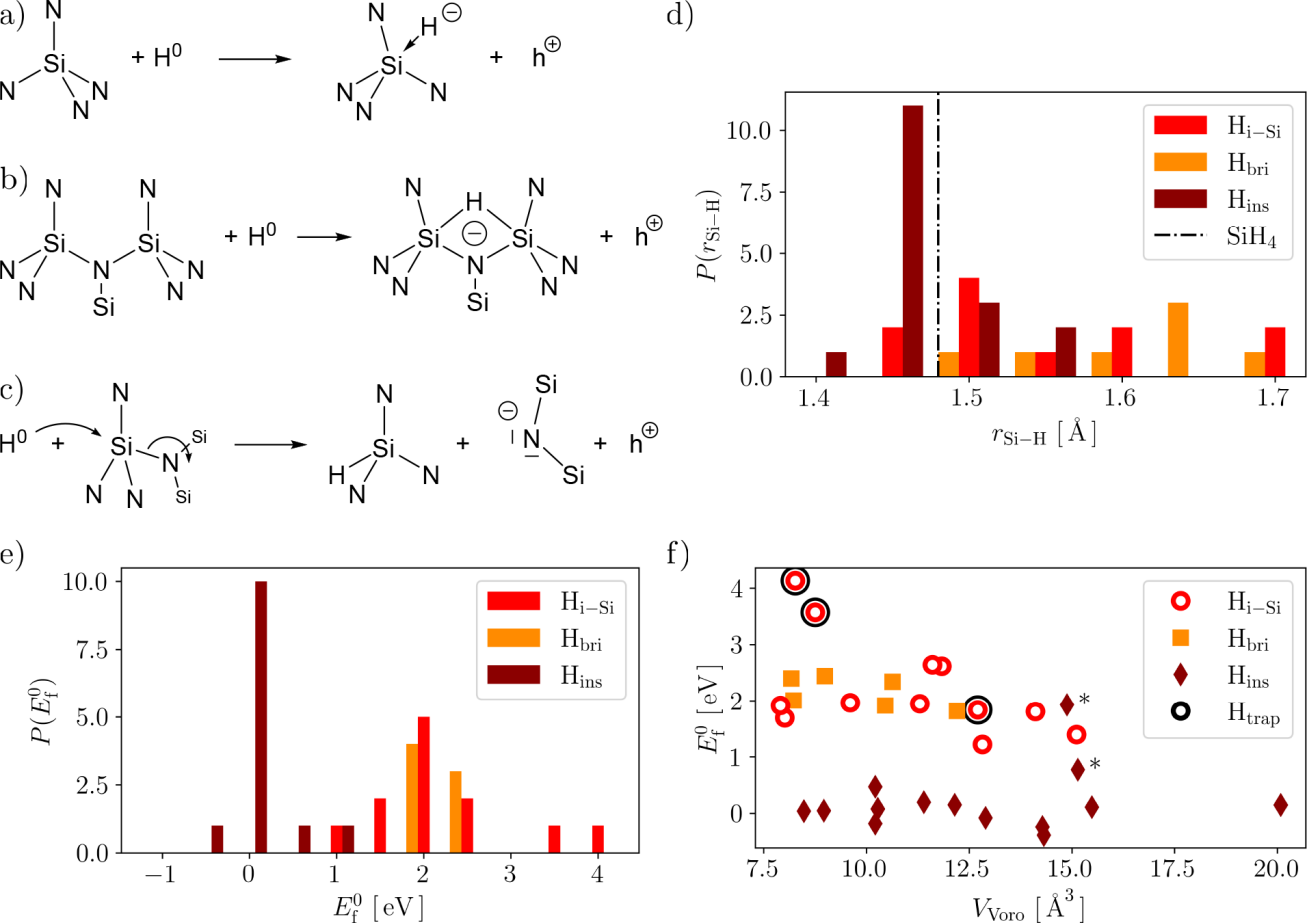
Schematic representation of Si–H defect configurations in the neutral charge state (*q* = 0) with a) H interstitial sites (H_i-Si_), b) bridging H sites (H_bri_), and c) H insertion sites (H_ins_). The electron deficit to form a Si–H bond is compensated for by taking an electron from the valence band, inducing a hole polaron (h^+^) at a site uncorrelated with the H defect. d) Distribution *P* of the Si–H bond length with H_i-Si_ (red), H_bri_ (orange), and H_ins_ configurations (dark red). For comparison, the Si–H bond length of silane (SiH_4_) is indicated by the dashed–dotted line.^61^ e) Distribution *P* of the formation energies *E*_f_^0^ (the same color scheme as in d). f) *E*_f_^0^ as a function of the Voronoi volume *V*_Voro_ for the configurations H_i-Si_ (hollow circles), H_bri_ (squares), and H_ins_ (diamonds). There are three cases marked with black circles (H_trap_), which have a notably different electronic structure causing H-induced trapping, as further discussed in the text.

The Si–H bond lengths spread over a range from 1.40 to 1.69 Å (Figure 3d), with the H_i-Si_ and H_bri_ configurations being significantly elongated compared to the Si–H bond length in silane (1.48 Å).^61^ On average, they measure 1.53 and 1.59 Å, respectively. The H_bri_ bonds are shifted to higher values as they are shared between adjacent Si atoms. In the H_i-Si_ and H_bri_ configurations, the coordination shell of the Si remains intact. In contrast, the H_ins_ sites show a dramatic elongation of one of the Si–N bonds with a mean separation of 2.75 Å post relaxation, while the Si–H bond relaxes to 1.47 Å on average, approximately the same length found in the silane (Figure 3d). The range of Si–H bond lengths is dictated by the local environment, impacting how the H is incorporated initially and subsequently how the defect center is able to relax. As schematically depicted in Figure 3c, H insertion occurs via backside insertion on the SiN_4_ tetrahedra so that the opposing Si–N bond is broken while the Si-center back projects. The relaxation bears similarities to the “puckering” described for oxygen vacancies in a-SiO_2_,^55,62^ albeit with some important differences as the relaxation drives the breaking of an Si–N bond and is restricted due to the lack of flexibility imparted by three-coordinate N-anions. This is in contrast to H-induced bond rupture found in SiO_2_ where insertion is driven by interaction with the O site, resulting in the hydroxylated E′ center (O_3_Si–OH + SiO_3_).^53,54^

In terms of formation energy *E*_f_^0^, the Si–H defects extend over a wide range between −0.39 and 4.13 eV (Figure 3e). The energy range can be further grouped by the incorporation modes as shown in Figure 3e,f: analogous to interstitial sites centered on nitrogen, H_i-Si_ scales with the local steric environment (*V*_Voro_), where regions with low steric repulsion (large *V*_Voro_) are favored over sterically crowded regions (small *V*_Voro_). Furthermore, in common with N–H centers, interaction with a neighboring Si atom results in stabilization of the H_bri_ configurations. It is interesting that there are two intermediate configurations identified, where the local environment frustrates the relaxation to H_ins_, resulting in an H–SiN_3_–N-type configuration (Figure 3f indicated with an asterisk). In addition and as indicated in Figure 3f by black circles, there are three H_i-Si_-like cases that are electronically distinct, as further analyzed below. In contrast, for H_ins_ sites a different picture emerges: *E*_f_^0^ sits in a tight range between −0.39 and 0.77 eV with the mean at 0.11 eV, with a small number of outliers at higher energy where the local geometry frustrates the relaxation. *E*_f_^0^ is largely independent of the local steric environment, being driven by the H insertion.

Focusing on the majority of the Si–H configurations, the corresponding +1 and −1 charge states turn out to leave the H-incorporation geometry unchanged, just as observed for the N-centered H defects above. The reason for this is illustrated by the DOSs shown in Figure 4a,b) for H_ins_ and H_bri_, respectively. The H states sit above the CBM (H_ins_) and below the VBM (H_bri_), resulting in an electrically inactive H center. The charge trapping in the system is mediated by h^+^ trapping on two-coordinate N as previously described.^58^ For H_ins_, the formation of the Si–H and creation of the two-coordinate N^0^ results in the relaxation of the h^+^ to the lowest-energy trap site. In H_i-Si_ and H_bri_, the H is bound to one or more Si, resulting in a H^–^ (quantified by a Mulliken charge of −0.1e) in line with the trend from the H^–^–cation interaction observed in oxides. The formation of H^–^ is driven by the H states sitting below the VBM: The neutral charge state Si + H^0^ yields Si ← H^–^ + h^+^ after charge relaxation (where ← symbolizes dative bonding). In each case, the hole is localized on the same two-coordinate N, which was the previously identified hole trapping site in the H-free case. Analogous to the N centers, charge trapping is thus dominated by a feature of pristine a-Si_3_N_4_ and rationalizing the very small spread of the CTLs shown in Figure 4d as a consequence.

**Figure 4:**
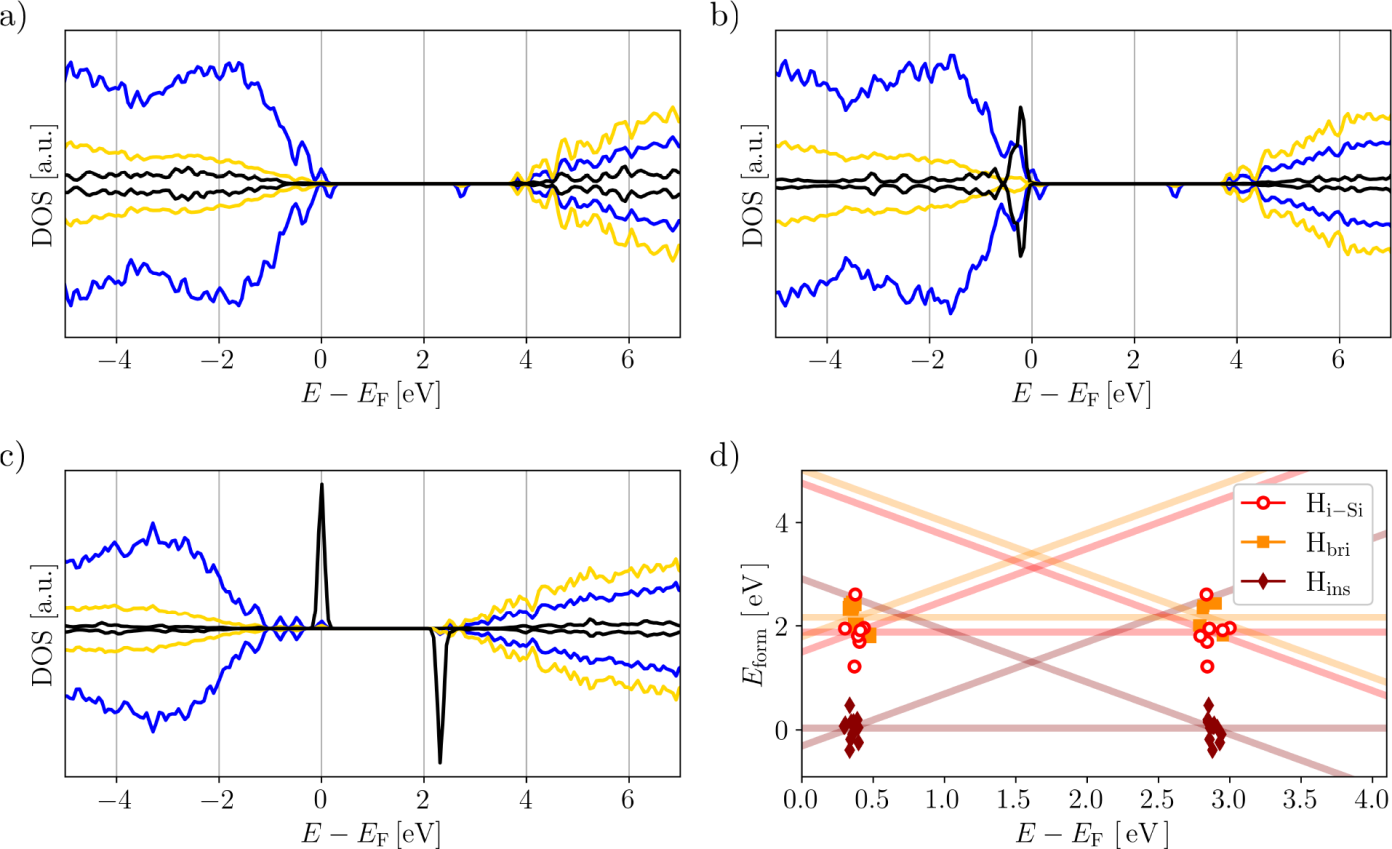
a–c) Projected electronic density of states (DOS) of Si (yellow), N (blue), and H (black) relative to the Fermi level *E*_F_. The DOS of H is amplified by a factor of 50 for visibility. a) and b) show DOSs for individual configurations representative of H_ins_ and H_bri_, respectively. c) The H_i-Si_ configuration shown here is exceptional, where no proper Si–H bond could be formed. This results in a single electron being localized on the H atom while not inducing a hole polaron in the bulk (as depicted in Figure 3a). d) Formation energies *E*_form_ as a function of the Fermi level *E*_F_ for the H_i-Si_, H_bri_, and H_ins_ defects in the +1, neutral, and −1 charge states (same color code as in Figure 3f). Solid lines mark the average over the entire ensemble together with the individual data points for the +1/0 (average 0.4 eV) and 0/–1 (average 2.9 eV) charge transition levels.

We now return to those H_i-Si_ configurations which are marked in Figure 3f by black circles. Based on their geometry, the H atom appears to interact with a single Si atom only in those cases. However, the representative DOS shown in Figure 4c and the charged counterparts of these configurations reveal something quite different, namely, that in all of those cases H-induced traps form. These traps are formally in the neutral charge state (H^0^) with the frontier states being both H in nature. This is the reason that they show negative-*U* (close to *U* = 0 eV) behavior with a charge-dependent structural relaxation of the incorporated H atoms (more details in section 6 of the Supporting Information), as extensively described for amphoteric hydrogen defects in wide band gap oxides.

H incorporation in a-Si_3_N_4_ is rich and notably different from what has been previously described for crystalline β-Si_3_N_4_, where the formation of negative-*U* amphoteric defect centers dominates, as also seen in a broad range of wide band gap oxides. Table 1 provides a schematic overview for a-Si_3_N_4_, including its charge dependence, which we discuss further in the following.

**Table 1:** Simplified Schematic Overview of Charge Relaxation for Both N- and Si-Centered H Defects for Different (Global) Charge States *q*^a^

*q*	N–H		Si–H	
	unrelaxed	relaxed	unrelaxed	relaxed
+1	N + H^+^	N → H^+^	Si + H^+^	Si ← H^–^ + 2 h^+^
0	N + H^0^	N → H^+^ + e^–^	Si + H^0^	Si ← H^–^ + h^+^
–1	N + H^–^	N → H^+^ + 2 e^–^	Si + H^–^	Si ← H^–^

a For each defect center, the two subcolumns describe the unrelaxed and relaxed charge configurations. For the latter, e^–^ denotes an intrinsic electron polaron, and h^+^ denotes an intrinsic hole trap. The N/Si–H bonds are presented as dative bonds (→/←) to describe the rearrangement of electrons upon H incorporation.

In agreement with previous work,^33,38,59^ H can passivate dangling bonds, removing those defects from the band gap. These defects are always the lowest-energy configuration and in essence represent a healing of the network via the removal of undercoordinated centers. Their formation energies are negative with the chemical potential convention employed here and are thus favored regardless of the starting geometry. It is important to note that while energetically favored, the concentration of passivation sites is limited by the number of dangling bonds present in the pristine cell used for this study.

The insertion of H across strained Si–N bonds is found to provide a route for H-induced defect formation and disruption of the amorphous network. It constitutes an important atomistic mechanism for the experimentally observed H-induced softening of a-Si_3_N_4_.^23,43,44^ While having some similarities with the H-induced bond rupture seen in SiO_2_, there are some important differences, with Si–H formation being favored as opposed to N-centered H defects. Both in our study and within a realistic a-Si_3_N_4_ film, H insertion is naturally limited by the number of strained precursor sites. The further implication is that the strain present in the film as a result of the substrate or growth conditions will directly impact the H_ins_ concentration by influencing the number of precursor sites. The same argument can be extended to the distribution of precursor sites within a given film, with more strained environments found in close proximity to the a-Si_3_N_4_–substrate interface(s) within a device stack.

A majority of H defects interact with N/Si with intact coordination shells and sit in a broad range of formation energies with the H_i-N_ (*E*_f_^0^ = 1.81 eV) centers typically lower in energy than H_i-Si_ and H_bri_ (*E*_f_^0^ = 2.18 eV). The range of energy is largely dictated by the steric environment of the reference atom in the pristine state before H incorporation. Perhaps most intriguingly, the CTLs sit in a narrow range for each of the main classes of H defects for both centers (H_i-N_, H_i-Si_, and H_ins_). This very small spread of the CTLs across a range of defect configurations in stark contrast to their formation energetics seems puzzling at first glance. An examination of the nature of the defect states reveals the reason: in each case, the H configuration is independent of the charge state. This is driven by the H states sitting deep within the valence (and conduction) bands, thereby playing no active role in charge trapping. Instead, they merely act as carriers of an initial electron or hole localizing in a charge trap state predetermined by pristine a-Si_3_N_4_, i.e., at the h^+^ and e^–^ trap sites we have identified in our previous work.^58^ This is schematically summarized in Table 1.

We now compare H incorporation in a-Si_3_N_4_ to that of the much more widely studied oxide system. Here, H forms amphoteric defects whose charge states are dictated by the Fermi energy with the (+/−) CTLs sitting approximately 4.25 eV below the vacuum level.^48,51^ In contrast, H incorporation in a-Si_3_N_4_ is quite different, where the localized nature of the band edges and the propensity for the system to trap electrons and holes result in a charging of the a-Si_3_N_4_ dielectric layer. In common with a-SiO_2_, H in a-Si_3_N_4_ is found to facilitate the breaking of strained bonds. However, the mechanisms are quite different: rather than creating an analog of the hydroxylated E′,^53^ H preferentially adds to the Si site in the first instance. It is equally important to note that in the case of a-SiO_2_ the charge trapping occurs at the center where the bond breaks, whereas in a-Si_3_N_4_ the charge is located on a trap site unrelated to the site of H insertion.

At this juncture, it is important to raise two questions that go beyond the scope of this work. First, how is H incorporation affected in Si- and N-rich Si_*x*_N_*y*_? This is important because tuning the stoichiometry is used to achieve the application-specific performance of the material. In cases where deviations from stoichiometric a-Si_3_N_4_ are small, it would be reasonable to expect behavior similar to that described here. However, when the deviations are significant, new structural motifs might be introduced that are not captured by the present study. Second, how do the nature of the intrinsic trap sites and the range of levels previously described^58^ affect H incorporation? Both of these questions together with the impact of increasing the H concentration are currently under investigation.

In summary, our investigation of H defect centers in a-Si_3_N_4_ uncovers the multifaceted role of H. While hydrogen has been known to passivate dangling bonds, thereby enhancing the electronic and optical properties playing the role of “Jekyll”, beyond passivation, its “Hyde” also emerges: incorporated H atoms instigate Si–N bond disruptions, especially in strained regions characterized by a distorted Si coordination environment and at least one elongated Si–N bond. It is noteworthy that a majority of hydrogen-associated states, whether from NH or most SiH configurations, reside within the valence and conduction bands, rendering them electronically inert. Charge trapping is predominantly linked to intrinsic traps for NH defects and N centers (or two-coordinate N) in the case of SiH defects, although a few high-energy H configurations act as exceptions. The identification of an intrinsic electron bipolaron in the −1 charge state for NH defects, which induces a significant relaxation in the amorphous network, requires further study. Importantly, this study bridges the understanding between H behavior in a-SiO_2_ and a-Si_3_N_4_ while emphasizing some important differences. It is plausible to infer, and left for future work to be confirmed, that analogous behavior could be seen in materials characterized by band edges that show a degree of localization.

## Computational Methods

Fermi energy and charge-dependent defect formation energies *E*_form_(*E*_F_; *q*) for the incorporation of hydrogen into a-Si_3_N_4_ were calculated using the standard formalism of Zhang and Northrup^63^ based on density functional theory (DFT) at the hybrid functional level. The chemical potential of H has been taken as  H_2_. In the following, defect formation energies for the incorporation of hydrogen in the neutral charge state (*E*_f_^0^ = *E*_form_(*E*_F_;0)) are generally referred to as *the* formation energies. By convention, the charge transition level (CTL) is defined as the Fermi energy at which the formation energy of two charge states is equal (*E*_form_(*q*) – *E*_form_(*q*′) = 0). Finite-size corrections for the charged systems are performed using the Lany–Zunger correction scheme.^64^ All DFT calculations were performed spin-polarized with the CP2K^65^ code using the HSE06^66,67^ exchange-correlation functional with the auxiliary density matrix method (ADMM).^68^ The DZVP-SR-MOLOPT^69^ family of basis sets was employed to describe the valence electrons together with GTH pseudopotentials^70−72^ for the core electrons. More computational details are provided in the Supporting Information.

The problem of structure sampling was explored at length in our previous work as it represents a vital consideration for amorphous systems.^58^ A single a-Si_3_N_4_ cell is selected to allow the problem of site sampling to be untangled from the broad range of intrinsic trap sites previously described. To achieve this, the previous statistical sampling scheme is extended to the consideration of H defects, capturing the variety of geometries of the host lattice. These results are included in the Supporting Information and result in the selection of 60 seed sites for H incorporation, with 25 (35) originally centered at Si (N), respectively.

## Data Availability

All structures generated in this study are available via zenodo.org (10.5281/zenodo.10054617), together with input parameters and specifications for the compilation of the CP2K package.

## Supporting Information Available

More computational details; hydrogen incorporation in β-Si_3_N_4_; properties of the pristine cell; statistical sampling of hydrogen incorporation sites; additional characterization of structural effects; and hydrogen-induced trap sites (PDF)
