# Supplementary material for: From Jekyll to Hyde and Beyond: Hydrogen’s Multifaceted Role in Passivation, H-Induced Breakdown, and Charging of Amorphous Silicon Nitride
Source: J Phys Chem Lett. 2024 Jan 18;15(3):840–8. doi: 10.1021/acs.jpclett.3c03376 (PMC10823530; doi:10.1021/acs.jpclett.3c03376)
Supplement: Supplementary file 1 — jz3c03376_si_001.pdf [file jz3c03376_si_001.pdf]

Supporting Information: From Jekyll to Hyde and Beyond: Hydrogen's Multifaceted Role in Passivation, H-Induced Breakdown, and Charging of Amorphous Silicon Nitride

Jonathon Cottom,[†] Lukas Hückmann,[†] Emilia Olsson,[‡,¶] and Jörg Meyer[*,†]

†*Leiden Institute of Chemistry, Gorlaeus Laboratories, Leiden University, P.O. Box 9502, 2300 RA Leiden, The Netherlands*

‡*Advanced Research Center for Nanolithography, Science Park 106, 1098 XG Amsterdam, The Netherlands*

¶*Institute for Theoretical Physics, University of Amsterdam, Postbus 94485, 1090 GL Amsterdam, the Netherlands*

E-mail: j.meyer@chem.leidenuniv.nl

Contents

1 More Computational Details S-3

2 Hydrogen Incorporation in β-Si_3N_4 S-6

3 Properties of the Pristine Cell S-8

3.1 Ensemble Properties S-8

3.2 Polaronic Charge Trapping S-9

4 Statistical Sampling of Hydrogen Incorporation Sites S-10

5 Additional Characterization of Structural Effects S-11

5.1 X−H Bond Length Distribution S-11

5.2 First Coordination Shell of the N−H Center S-12

5.3 Trap Site Associated with the N−H Center S-14

5.4 First Coordination Shell of the Si−H Center S-16

5.5 Trap Site Associated with the Si−H Center S-19

6 Hydrogen-Induced Trap Sites S-21

References S-22

1 More Computational Details

Charge-dependent defect formation energies for incorporation of hydrogen into a-Si_3N_4 are calculated using the standard formalism of Zhang and Northrup[S1]

$$E_{\text{form}}(E_{\text{F}};q) = E_{\text{a-Si}_3\text{N}_4\,+\,\text{H}}(q) - E_{\text{a-Si}_3\text{N}_4} - \tfrac{1}{2}E_{\text{H}_2} + qE_{\text{F}} + E_{\text{corr}}(q) \quad . \tag{1}$$

$E_{\text{a-Si}_3\text{N}_4}$ and $E_{\text{a-Si}_3\text{N}_4\,+\,\text{H}}(q)$ are total energies from density functional theory (DFT, *vide infra*) calculations of the hydrogen-free cell and one with hydrogen added at a particular site in charge state q, respectively. The chemical potential of H is referenced to $\frac{1}{2}E_{\text{H}_2}$, i.e., half of the total energy of an H_2 molecule. Assuming that electrons from the Fermi level E_{F} act as charge reservoir maintaining the proper charge balance, qE_{F} is the associated reference chemical potential. $E_{\text{corr}}(q)$ corrects for artificial (electrostatic) charge interactions of periodic charge images due to finite-sized cells in the DFT calculations, which in this work are performed according to the correction scheme suggested by Lany and Zunger[S2]

$$E_{\text{corr}}^{\text{LZ}} = \frac{\alpha\, q^2}{6\epsilon L}\left(1 - \frac{c_{\text{sh}}}{\epsilon}\right) \quad . \tag{2}$$

Here ϵ is the dielectric constant of a-Si_3N_4, α the Madelung constant, and L and c_{sh} describe the size and the shape of the DFT simulation cell. The dielectric (ϵ) of the reference cell was calculated in the linear response framework using the response of the electron density with respect to an applied field.

Defect formation energies for incorporation of hydrogen in the neutral charge state

$$E_{\text{f}}^{0} = E_{\text{form}}(E_{\text{F}};0) \tag{3}$$

are generally referred to as *the* formation energies. Finally, by convention, the adiabatic charge transition level (CTL) is defined as the Fermi energy $E_{\text{F}}^{\text{CTL}}$ at which the formation energies for hydrogen incorporation at a fixed site in two different charge states are identical,

i.e.

$$E_{\mathrm{form}}(E_{\mathrm{F}}^{\mathrm{CTL}}; q') - E_{\mathrm{form}}(E_{\mathrm{F}}^{\mathrm{CTL}}; q) = 0 \quad \text{with } q' = q - 1. \tag{4}$$

An ensemble of structures modeling amorphous Si_3N_4 had been generated via a MD melt-quench procedure, using the MG2 potential,[S3–S5] a cooling rate of $1\,\mathrm{K\,ps^{-1}}$, and the Nosé-Hoover thermostat and barostat[S6–S8] employing the LAMMPS code.[S9] This approach was selected as it has been previously shown to produce reliable amorphous models[S10] that have good agreement with both the experimental[S11–S13] and theoretical[S14–S17] structures. The full procedure along with the structure database and characterization, is included in our previous work.[S10] For the present study, a single 280 atom a-Si_3N_4 cell has been selected from this database as a starting point, such that the range of Si and N sites and intrinsic charge trapping energies are representative for the full structural ensemble of a-Si_3N_4. This is characterized further in the subsequent sections. This pristine, hydrogen-free cell forms the reference system, which allows H-incorporation and the interplay with the previously identified intrinsic electron traps[S10] to be treated in a consistent manner.

The problem of structure sampling was explored at length in our previous work as it represents a vital consideration in modeling amorphous systems.[S10] To ensure appropriate site sampling, the previous scheme is extended to the consideration of extrinsic defects. As further illustrated in Section 4 this results in a selection of 60 sites, 20 of which were originally centered on Si and 40 on N so that the selection is in accordance with the stoichiometry. For each of the reference sites, a Voronoi polyhedron is constructed centered on the reference atom and the H placed on the face with the largest area (at least 2.0 Å from all sites), to ensure steric repulsion of the initial configuration is minimized, along with any associated artifacts. The Voronoi volume of the reference atom has been determined by delineating its closest enclosed region within the amorphous network. This was achieved using perpendicular bisector planes between the reference atom and its neighbors to describe the Voronoi polyhedra for the reference atom. In practice, both the Voronoi polyhedra and the associated volume are calculated by taking the atomic positions, lattice vectors accounting

for periodic boundary conditions, and finally the Voronoi search was conducted using the SciPy library.[S18] The so-constructed precursor geometry with the H atom is then allowed to relax free of any constraints but within an effective "local cage" given by the surrounding amorphous network. Consequently, the final site of the H atoms is not necessarily the nearest neighbor to the original reference atom. This holds in particular during the relaxation for the different charge states at the DFT level. The electronic ground state of pristine a-Si_3N_4 is in the low-spin configuration, with 32 paired valence electrons per unit of Si_3N_4. Consequently, adding H^0 results in a total spin moment $\mu_s \neq 0$, and the localization of the additional (unpaired) electron can be analyzed with the help of atomic projections of the spin density in the same way as in our previous work.[S10] The structural changes both around the H-incorporation sites and the charge trap sites are conveniently characterized by the cone angle descriptor (ϕ) introduced by Hückmann et al.[S10]. Section 5 shows the results of this structural analysis, which shows that while the trap sites undergo pronounced relaxation with coordination polyhedra getting deformed, the H-incorporation site is hardly affected.

All DFT calculations were performed spin polarised using the CP2K[S19] code with the DZVP-SR-MOLOPT[S20] family of basis-sets to describe the valence electrons, and GTH-pseudopotentials[S21–S23] to describe the core electrons. An energy cutoff of 650 Ry and a relative cutoff of 60 Ry was used, including only the Γ-point for Brillouin zone sampling. These settings yield a convergence of 0.1 meV per formula unit. The convergence criteria for SCF energy was 10^{-7} eV for all calculations and for forces 0.005 eV $Å^{-1}$ for the cell and geometry optimizations. The simulation cell vectors and ion positions were fully relaxed using the quasi-Newton BFGS update scheme (ion positions only for the defect calculations). An initial optimization was carried out using the PBE functional,[S24,S25] which is further refined using HSE06[S26,S27] with the auxiliary density matrix method (ADMM)[S28] for production as it significantly reduces the computational cost.

2 Hydrogen Incorporation in β-Si_3N_4

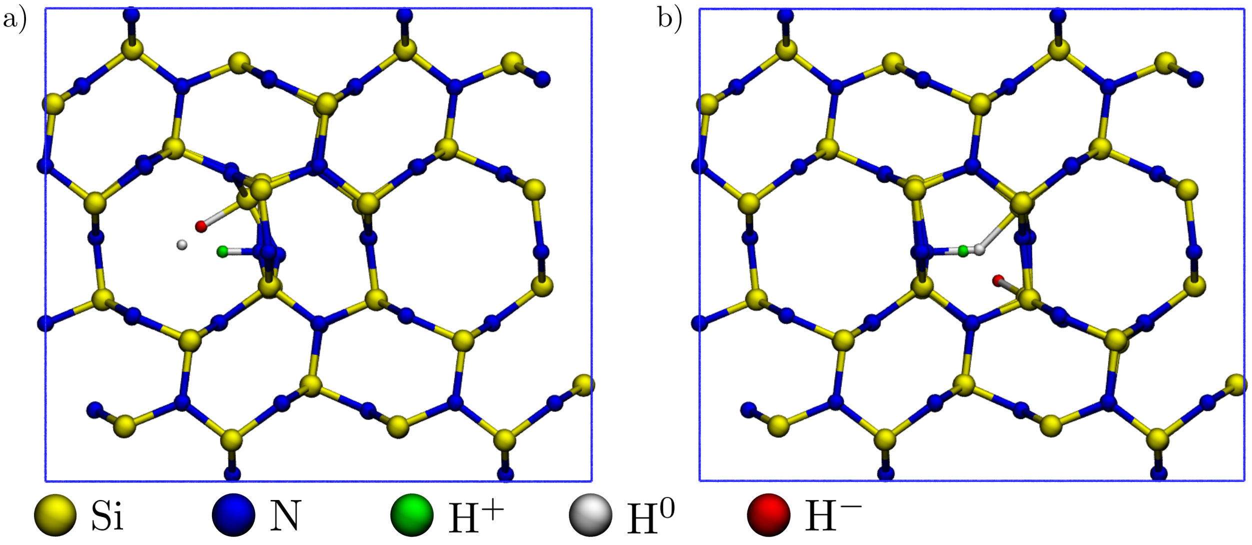

Figure S1: Schematic representation of the two H-incorporation sites in β-Si_3N_4 with a) H in the large tube and b) H in the small tube. Hydrogen atoms in the positive charge state are colored green, the ones in the neutral charge state are white, and the lines in the negative charge state are red. For computational ease, the structure is extended into an orthogonal box containing 280 atoms. The viewing direction of a) and b) is along the z-axis.

The incorporation of hydrogen in β-Si_3N_4 has previously been described by Grillo et al.[S29]. However, to ensure transferability between the crystal and the amorphous phase, their calculations have been repeated with the computational set-up described in Section 1. The structure of β-Si_3N_4 can roughly be described as layers in the xy-plane interlinked in z-direction with eight- and twelve-membered rings arranged in such a way that they form narrow and wide tubes along the z-axis, respectively. There are two distinct symmetry sites for nitrogen (Wyckoff letters $2b$ and $6c$), where only N($6c$) are spatially accessible for H. This leads to two distinct H-incorporation sites in the wide and in the narrow tube as shown in Figure S1a and b. In both cases, H in the neutral charge state (H^0, $q = 0$) does not interact with the lattice as it is placed in the middle of the cavity, maximizing the distance to neighboring atoms. In contrast, in the positive charge state (H^+, $q = +1$), H forms a bond with a nearby N atom resulting in a N−H bond length of 1.04 Å. Incorporation H^+ is favorable with a formation energy E_{form} of -1.12 eV for the narrow and -1.41 eV for the

wide tube, which is in accordance with steric effects dictated by the environment. In contrast, the negative charge state (H^-, $q = -1$) results in H binding to a silicon atom with the Si−H bond being 1.68 Å for the narrow and 1.51 Å for the wide tube. The frustrated Si−H bond, in combination with the detrimental fivefold coordination on the Si atom, leads to noticeably high formation energies 5.07 eV and 5.14 eV. Both sites share similar (+/−) charge transition levels at 3.10 eV (narrow) and 3.28 eV (wide, see Figure S2) and show negative-U behaviour with −0.86 eV and −0.45 eV.

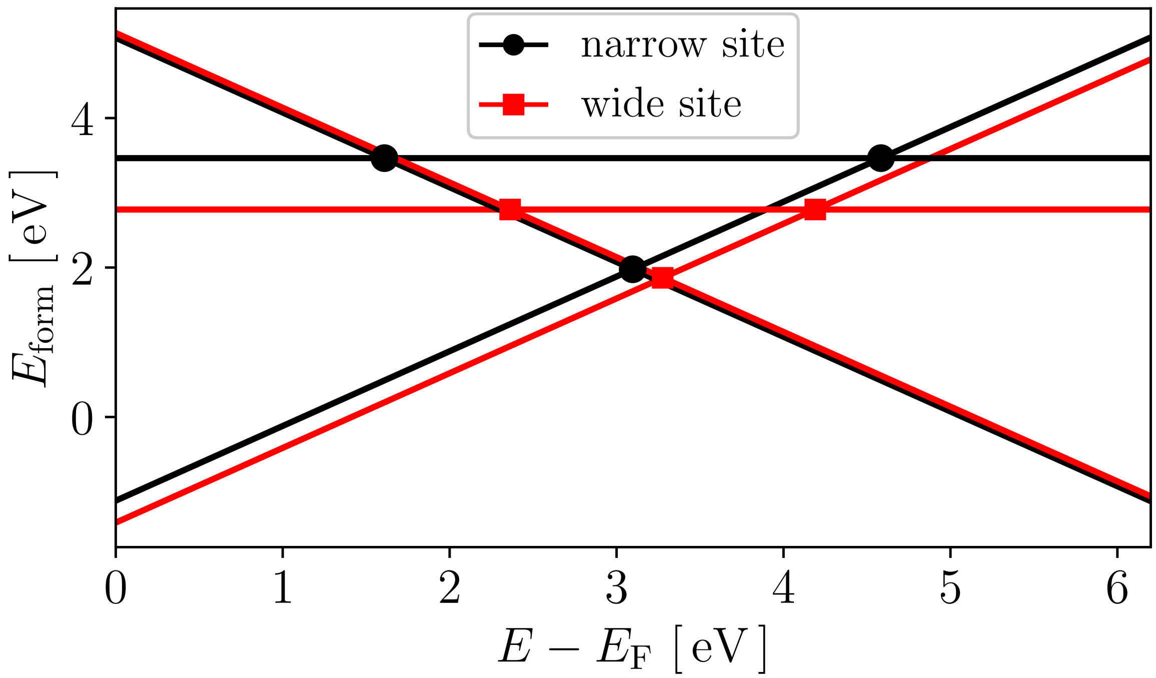

Figure S2: Formation energies E_{form} as a function of the Fermi level E_{F} for the hydrogen defect sites in the narrow (black, circles) and in the wide tubes (red, square) in the +1, neutral, and −1 charge states.

3 Properties of the Pristine Cell

3.1 Ensemble Properties

For this study, a single a-Si_3N_4 cell from the library generated in our previous work[S10] is selected to ensure a joint reference for all H insertions. For completeness, the properties of the pristine, hydrogen-free cell are listed in Section 3.1 together with the average values of the entire ensemble and – if available – literature data. The chosen sample appears to be average in all measures, so it is considered representative and appropriate to conduct hydrogen-insertion experiments.

Table S1: Properties of the pristine a-Si_3N_4 cell used in this study compared to ensemble averages from our previous study.[S10] Values for the density ρ, the coordination numbers CN, the band gap E_{bg}, and the trapping energies of holes E_{h^+} and electrons E_{e^-}. are obtained at the HSE06[S30,S31] level, whereas the bulk modulus B is from calculations with the MG2[S32] force field. Literature values are provided in addition if available.

Property	Unit	This study	Ensemble	Literature
ρ	$\mathrm{g\,cm^{-3}}$	2.9	2.9	2.6 to 3.0 [S33]
CN(Si)		4.00	3.96	3.70 [S12]
CN(N)		2.98[a]	2.87	2.78 [S12]
B	GPa	159	166	156 to 161 [S34]
E_{bg}	eV	4.10	4.43	4.77 [S35]
E_{h^+}	eV	−0.58	−0.35	−0.9 to −1.4 [S36]
E_{e^-}	eV	−0.53	−0.74	−1.2 to −1.7 [S36]
ϵ		8.45		7.0 to 10.5 [S14]
c_{sh}		-0.369	–	– [S2]
L	Å	14.72	–	–
Trap Type[b]		intr.	–	–

[a] ≙ four 2-coordinated N atoms; 160 N atoms in total.
[b] see Section 3.2.

3.2 Polaronic Charge Trapping

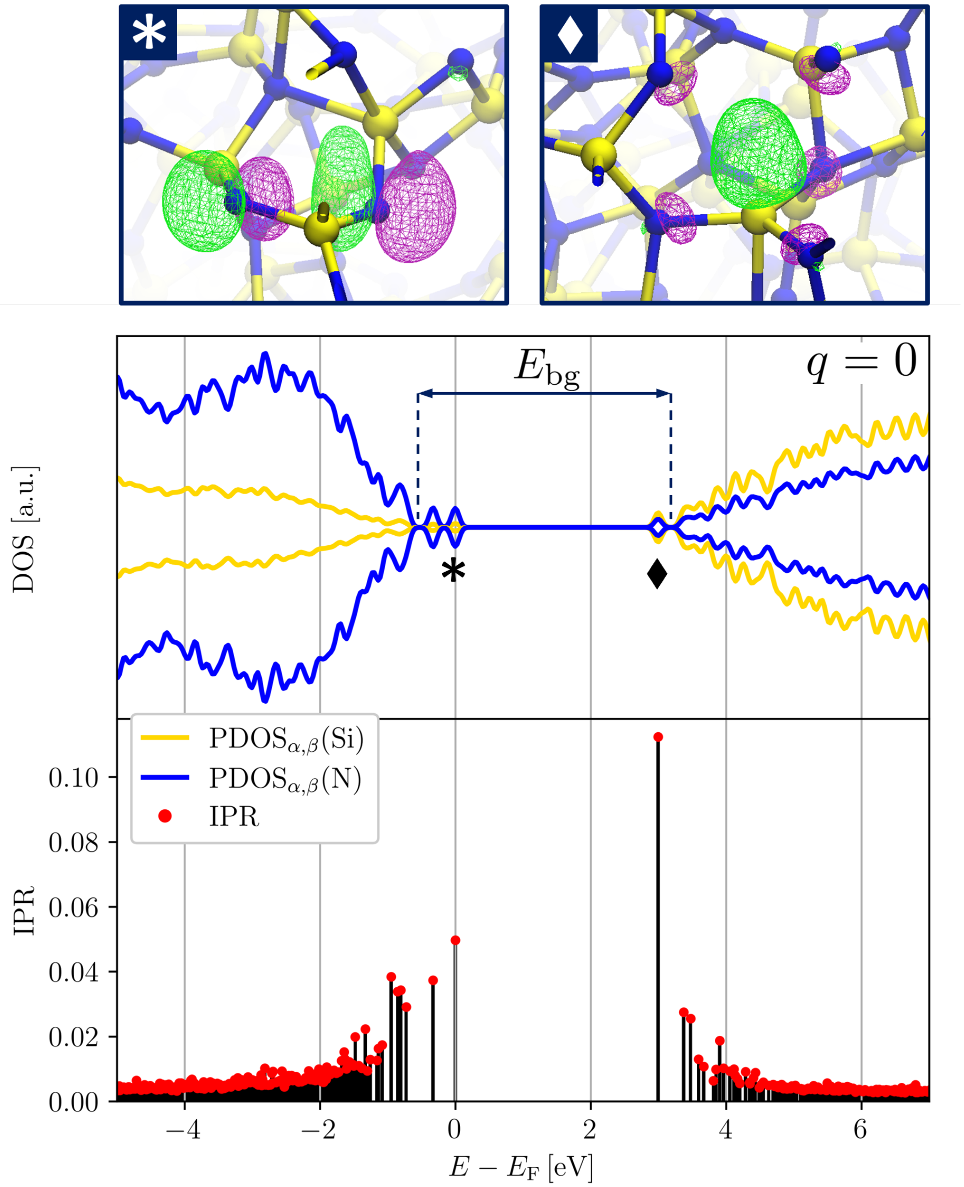

Figure S3: Upper panel: Illustration of the band edge states localized on two-coordinated nitrogen atoms above the VBM ($*$) and localized on distorted Si-tetrahedron below the CBM (♦). The isosurfaces of the spin density are set to $0.05\,e\,\text{Å}^{-3}$. Middle panel: The projected electronic density of states (DOS) of Si (yellow) and N (blue) in a single neutral a-Si_3N_4 configuration relative to the Fermi level E_F. The band gap E_{bg} is measured by excluding the localized states labeled with $*$, ♦. Lower panel: The corresponding inverse participation ratio for all eigenstates with $-5\,\text{eV} < E - E_F < 7\,\text{eV}$. All data is provided at HSE06 level. For more details, see Hückmann et al.[S10].

4 Statistical Sampling of Hydrogen Incorporation Sites

Hybrid-DFT calculations at, i.e., HSE06 level[S30,S31] are necessary to describe the electronic structure correctly, including band gaps and localized mid-gap states.[S37–S39] However, its computational cost limits its large-scale applicability. Instead of inserting H at all 280 sites, the sampling is reduced to 60 sites in the +1, 0, and −1 charge state each. To maintain statistical significance, the HSE-optimization set is selected by the scheme presented in.[S10] The only difference is the selection criterion being the cone angle ϕ as it offers a site-centered description capturing local symmetry and symmetry-breaking. The 60 sites are chosen so that 25 are centered on a Si atom and 35 on an N atom to approximately resemble the stoichiometry of 3:4. Figure S4 shows the resulting selection, which captures the parent distribution sufficiently.

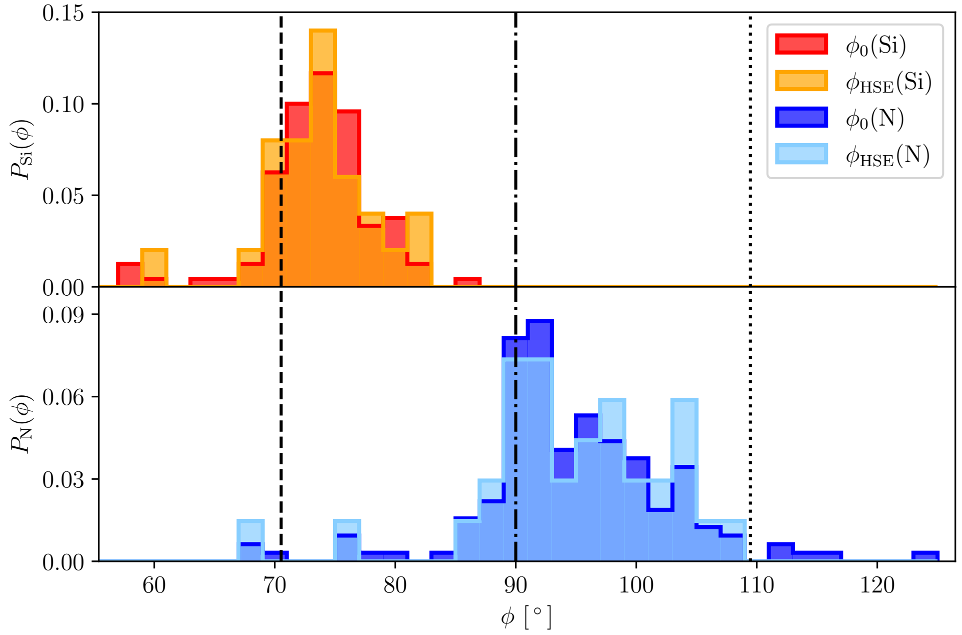

Figure S4: Cone angle distributions for all 280 sites ($P(\phi_0)$) and the 60 sites selected in this study for hydrogen incorporation at HSE06 level ($P(\phi_{\mathrm{HSE}})$). The distributions for silicon-centered sites are given in red/orange in the top panel, and those for nitrogen-centered are given in blue/cyan in the bottom panel. Vertical lines mark reference values for cone angles corresponding to an ideal tetrahedron (dashed), trigonal plane (dash dotted) and a trigonal pyramid (dotted).

5 Additional Characterization of Structural Effects

5.1 X−H Bond Length Distribution

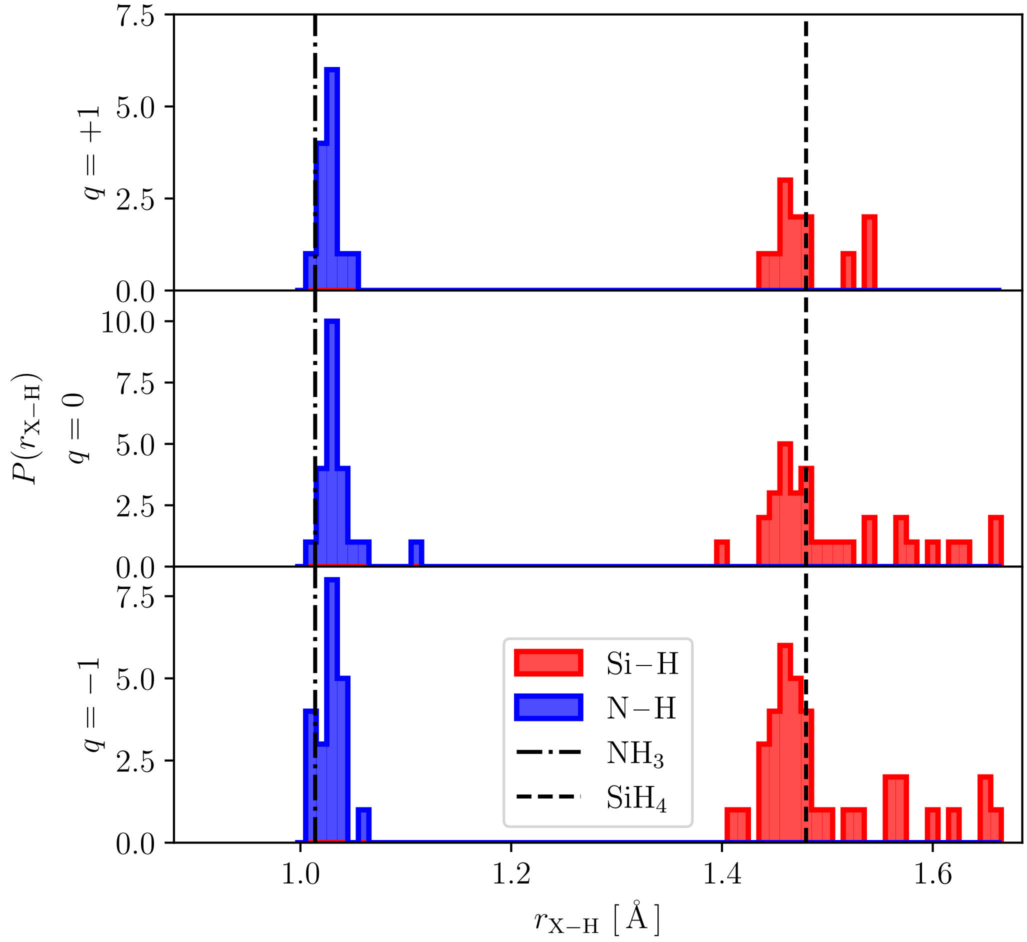

Figure S5: Distribution P of the X−H bond length $r_{\mathrm{X-H}}$ with $\mathrm{X} \in \{\mathrm{Si}, \mathrm{N}\}$ for the positive ($q = +1$, upper panel), the neutral ($q = 0$, middle panel), and the negative charge state ($q = -1$, lower panel). For comparison, literature values for the Si−H bond length in silane SiH_4[S40] and the N−H bond length in ammonia NH_3[S41] are given as the dashed and the dashed-dotted line, respectively.

5.2 First Coordination Shell of the N−H Center

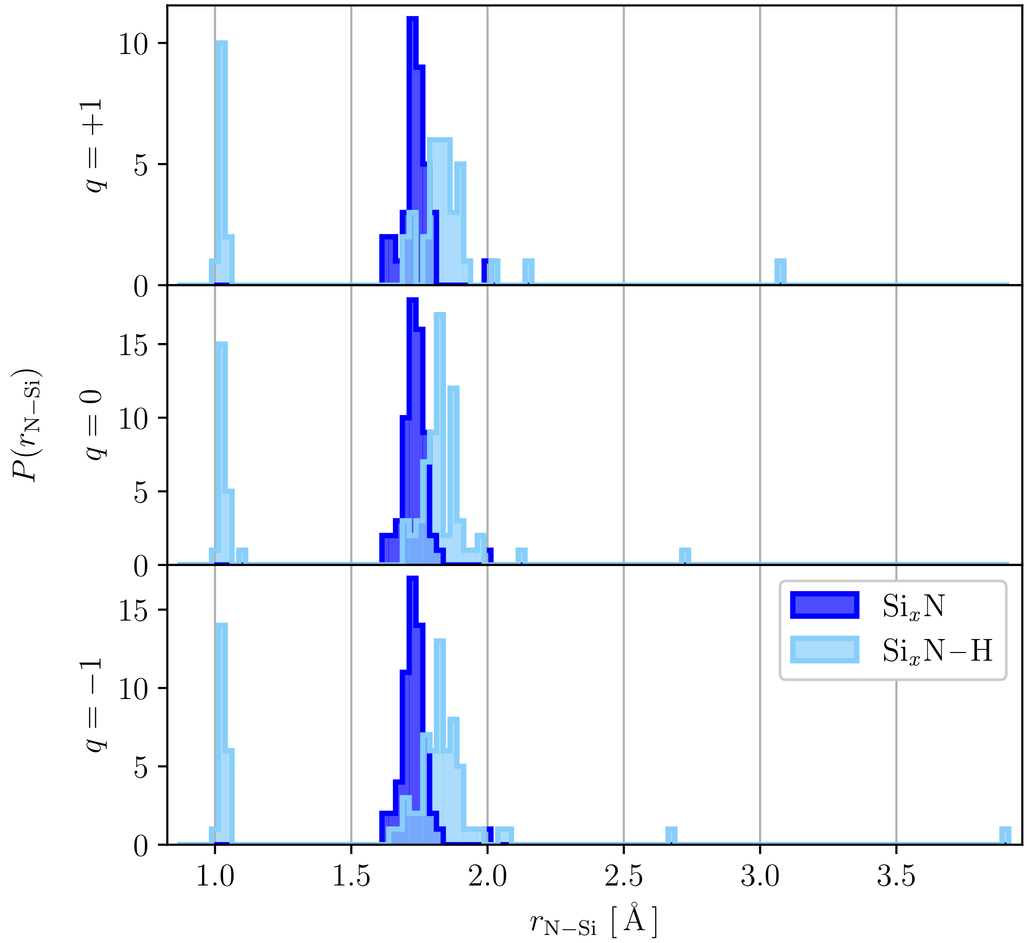

Figure S6: Distribution P of the N−Si bond lengths $r_{\mathrm{N-Si}}$ of the first coordination shell of the host N atoms before (dark blue) and after the H insertion (light blue) for the positive ($q = +1$, upper panel), the neutral ($q = 0$, middle panel), and the negative charge state ($q = -1$, lower panel).

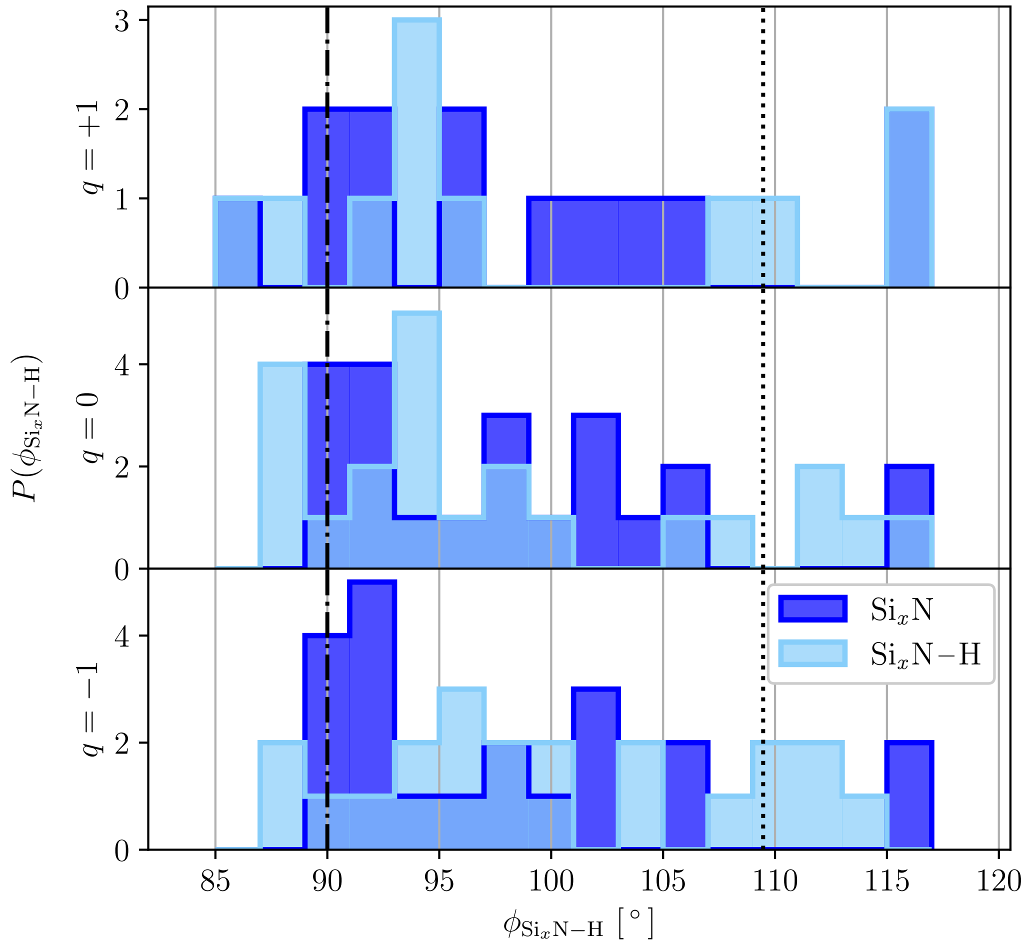

Figure S7: Distribution P of the cone angles $\phi_{\mathrm{Si_xN-H}}$ of the $\mathrm{Si_xN{-}H}$ centers before (dark blue) and after the H insertion (light blue) for the positive ($q = +1$, upper panel), the neutral ($q = 0$, middle panel), and the negative charge state ($q = -1$, lower panel). The cone angles of the trigonal plane (dash-dotted line), and of the trigonal pyramid (dotted line) are given as references.

5.3 Trap Site Associated with the N−H Center

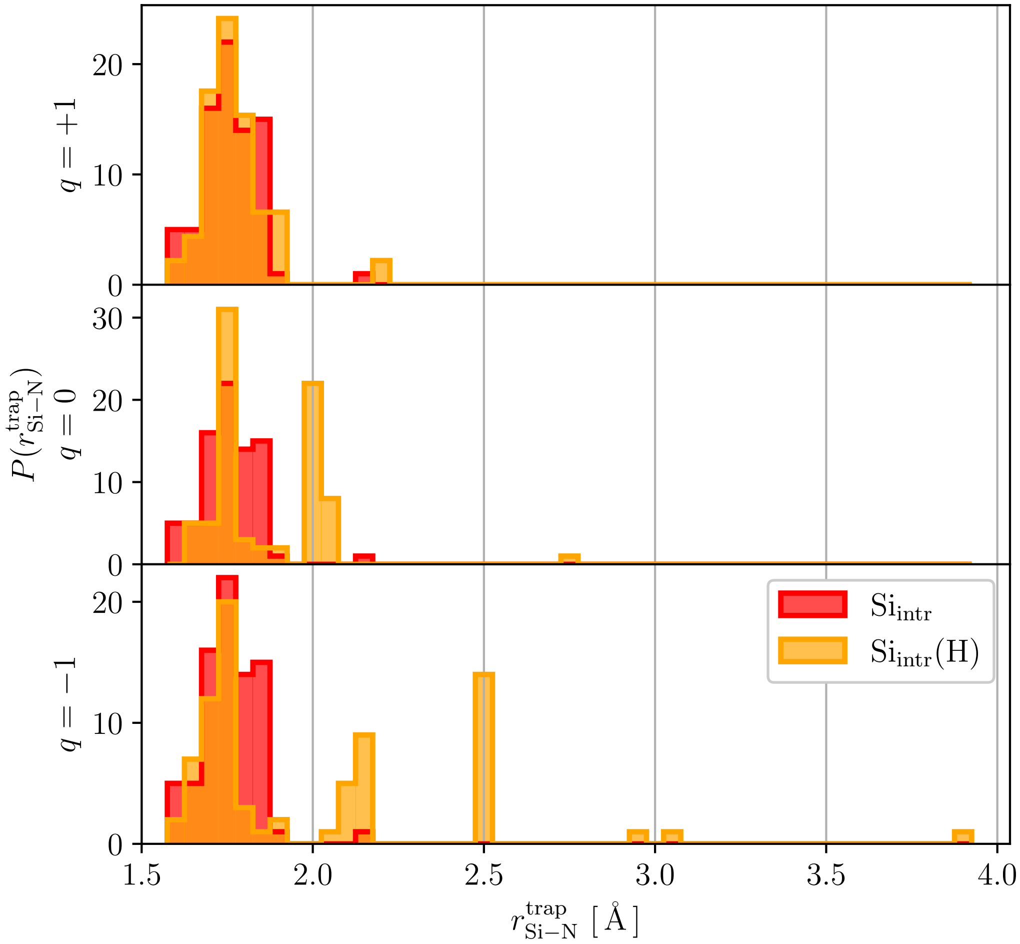

Figure S8: Distribution P of the N−Si bond lengths $r_{\mathrm{Si-N}}$ of the first coordination shell of the trap sites induced by H insertion before (red) and after the H insertion (orange) for the positive ($q = +1$, upper panel), the neutral ($q = 0$, middle panel), and the negative charge state ($q = -1$, lower panel).

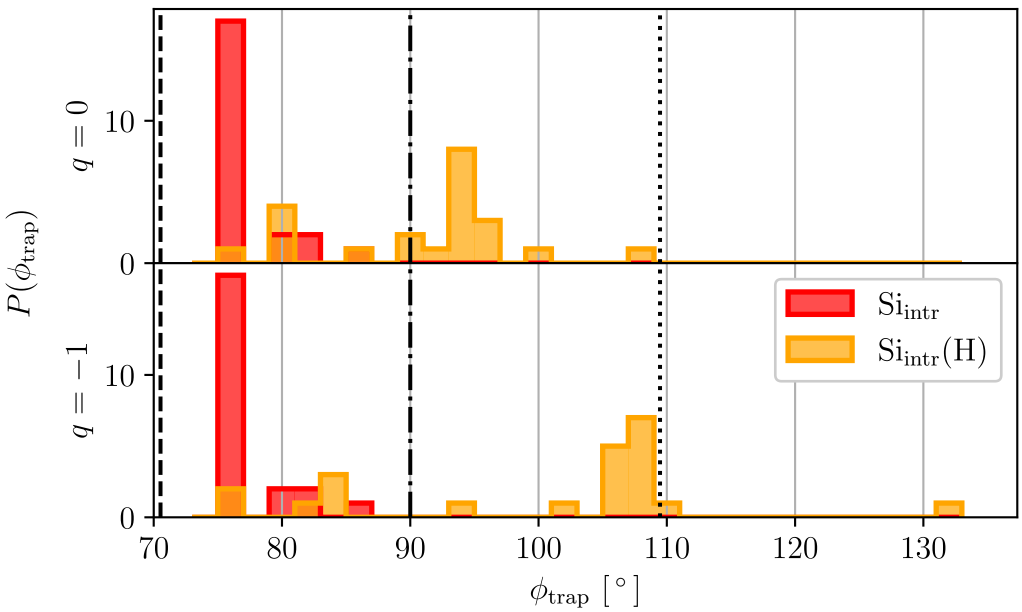

Figure S9: Distribution P of the cone angles ϕ_{trap} of the trap sites induced by H insertion before (red) and after the H insertion (orange) for the positive ($q = +1$, upper panel), the neutral ($q = 0$, middle panel), and the negative charge state ($q = -1$, lower panel). The cone angles of the trigonal plane (dash-dotted line), and of the trigonal pyramid (dotted line) are given as references.

5.4 First Coordination Shell of the Si−H Center

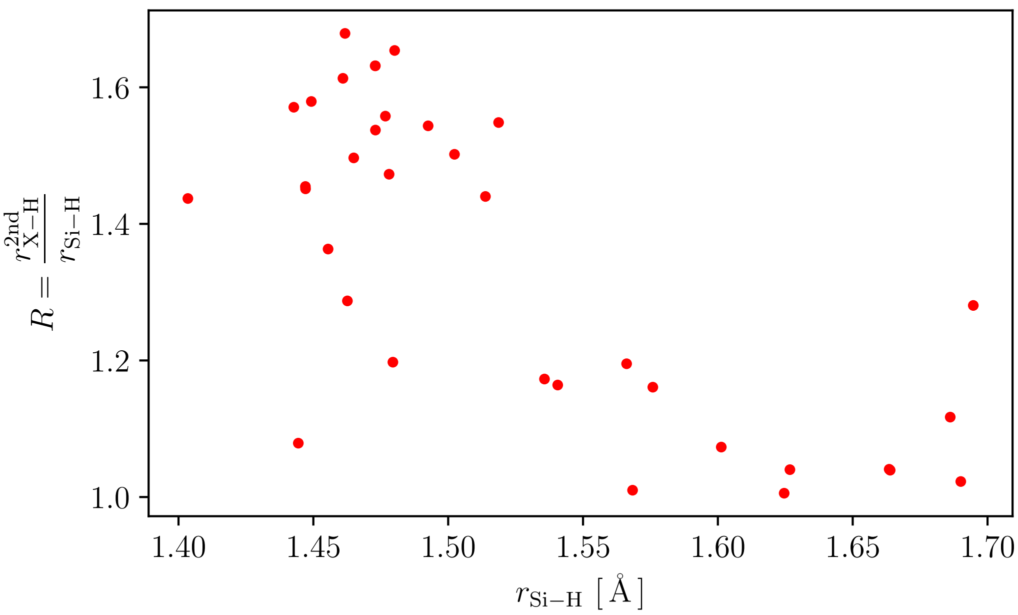

Figure S10: The ratio $R = \frac{r^{2nd}_{X-H}}{r_{Si-H}}$ of the second next neighbor's distance r^{2nd}_{X-H} over the next neighbor's distance r_{Si-H} of hydrogen as a function of r_{Si-H}.

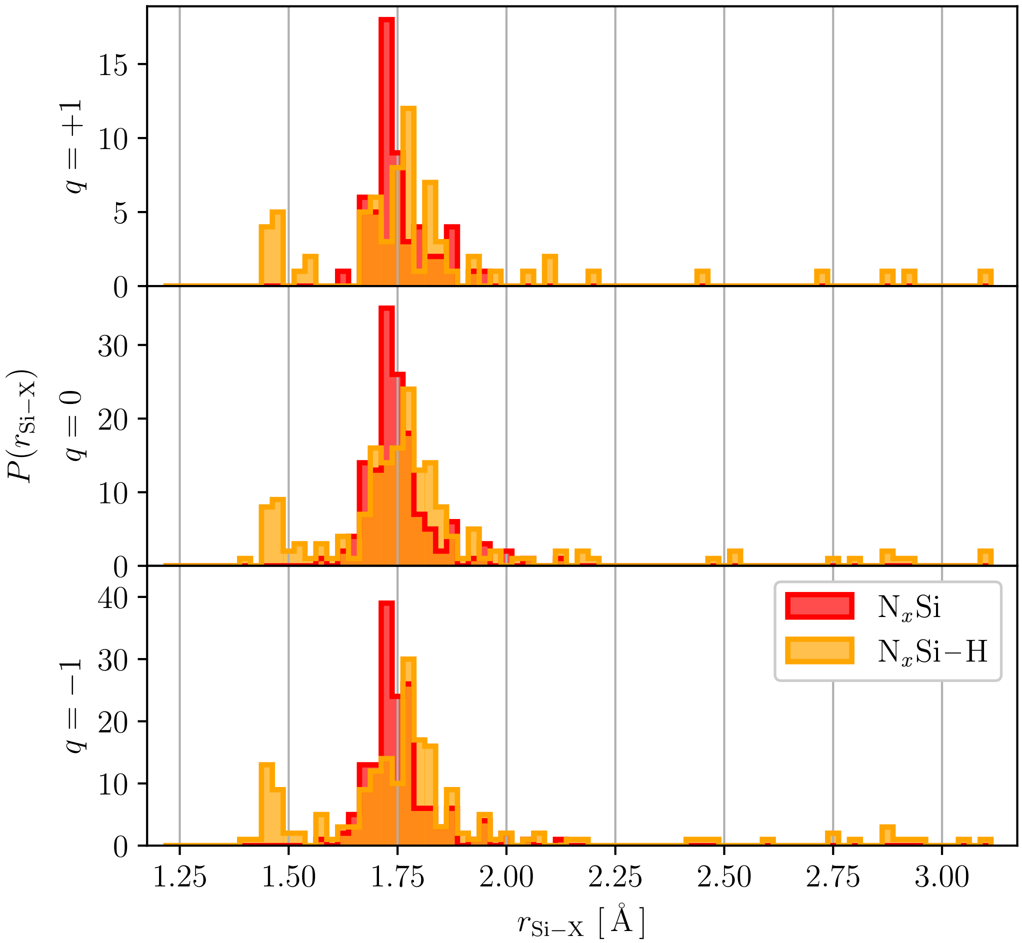

Figure S11: Distribution P of the Si−N bond lengths $r_{\mathrm{Si-N}}$ of the first coordination shell of the host Si atoms before (red) and after the H insertion (orange) for the positive ($q = +1$, upper panel), the neutral ($q = 0$, middle panel), and the negative charge state ($q = -1$, lower panel).

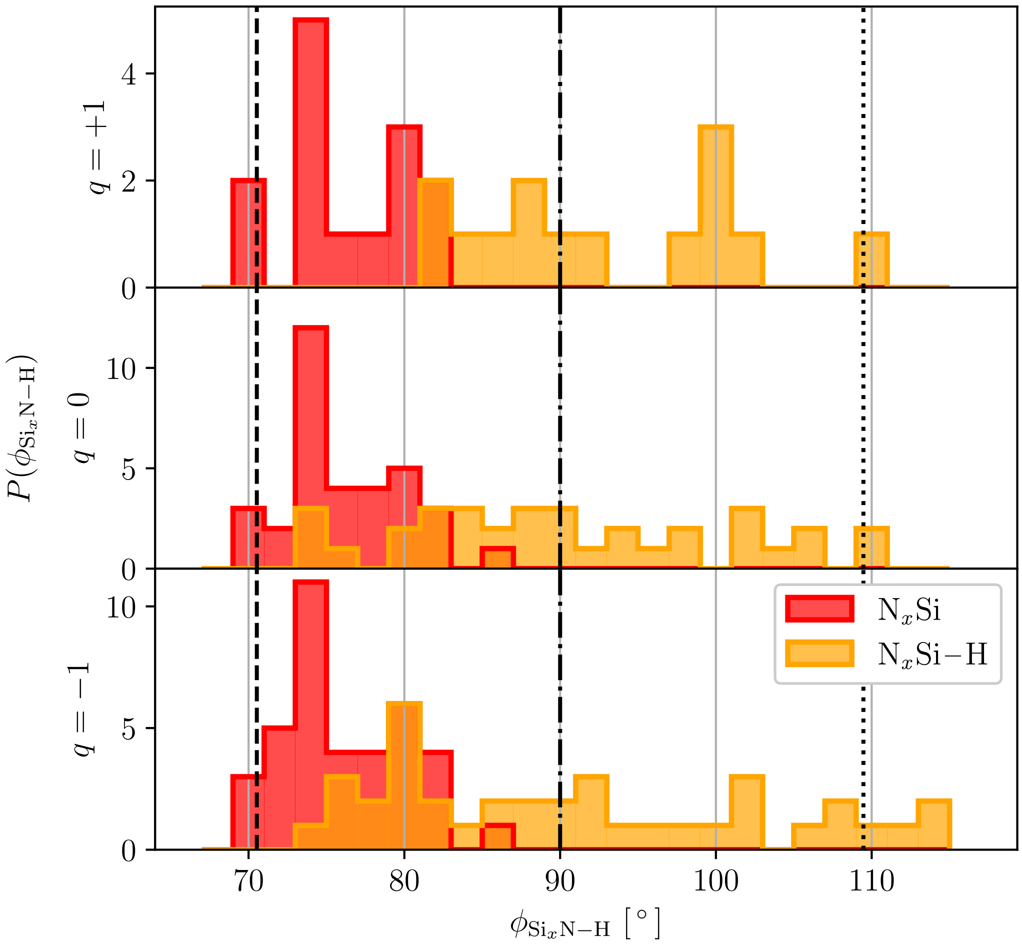

Figure S12: Distribution P of the cone angles $\phi_{\mathrm{N_xSi-H}}$ of the $\mathrm{N_xSi{-}H}$ centers before (red) and after the H insertion (orange) for the positive ($q = +1$, upper panel), the neutral ($q = 0$, middle panel), and the negative charge state ($q = -1$, lower panel). The cone angles of the trigonal plane (dash-dotted line) and of the trigonal pyramid (dotted line) are given as references.

5.5 Trap Site Associated with the Si−H Center

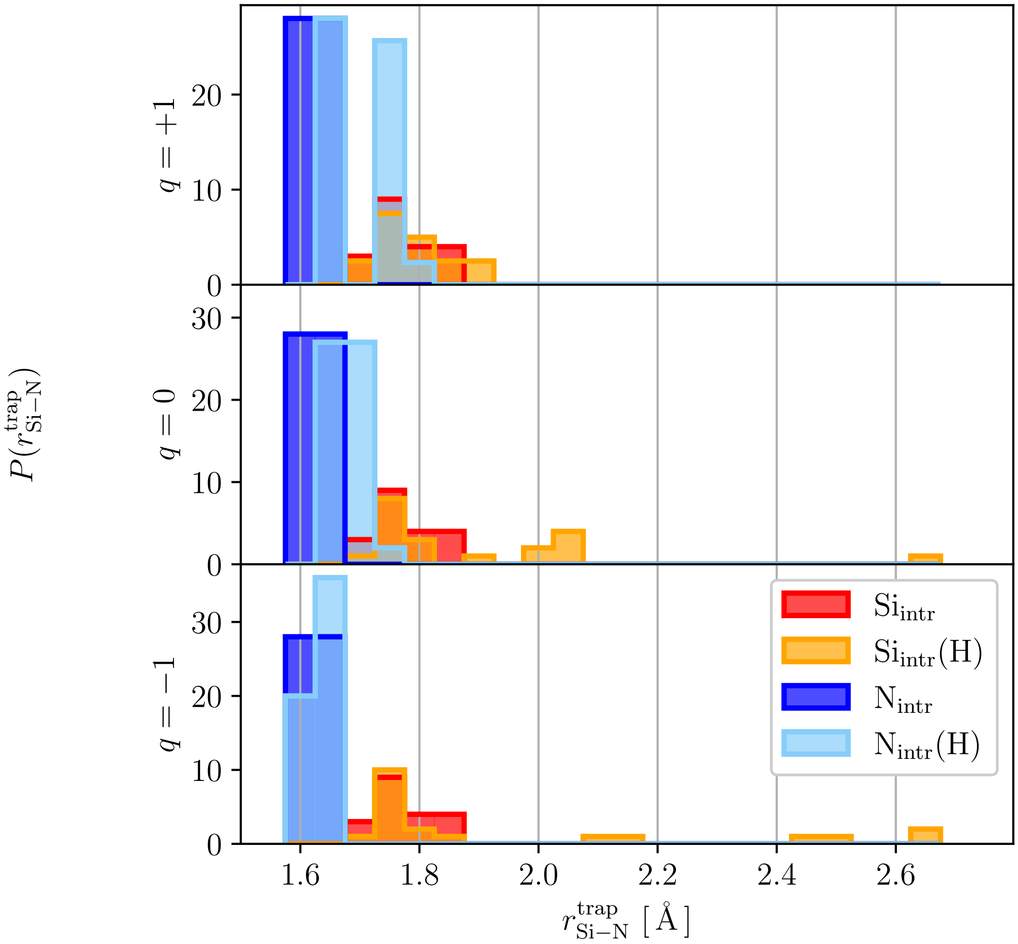

Figure S13: Distribution P of the N−Si bond lengths $r_{\mathrm{N-Si}}$ of the first coordination shell of the trap sites induced by H insertion before (blue) and after the H insertion (cyan) for the positive ($q = +1$, upper panel), the neutral ($q = 0$, middle panel), and the negative charge state ($q = -1$, lower panel).

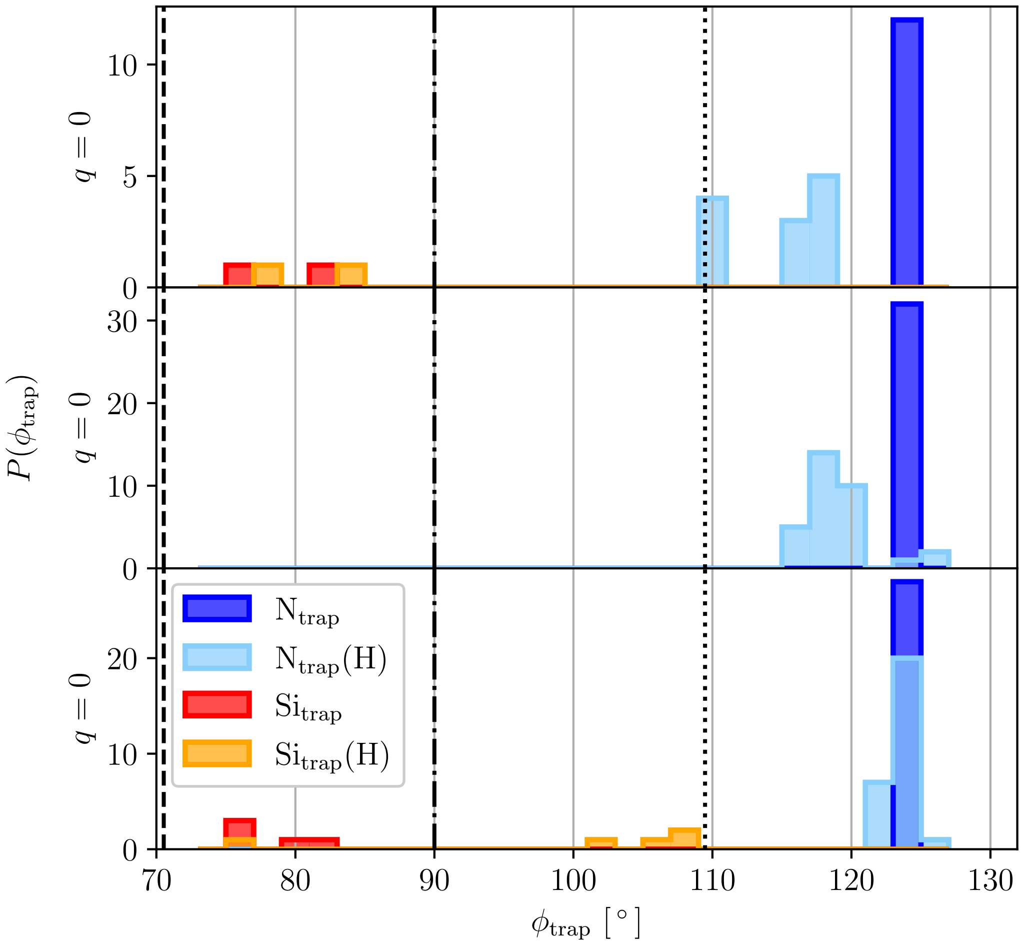

Figure S14: Distribution P of the cone angles ϕ_{trap} of the trap sites induced by H insertion before (red/dark blue) and after the H insertion (orange/cyan) for the positive ($q = +1$, upper panel), the neutral ($q = 0$, middle panel), and the negative charge state ($q = -1$, lower panel). The cone angles of the trigonal plane (dash-dotted line), and of the trigonal pyramid (dotted line) are given as references.

6 Hydrogen-Induced Trap Sites

a)

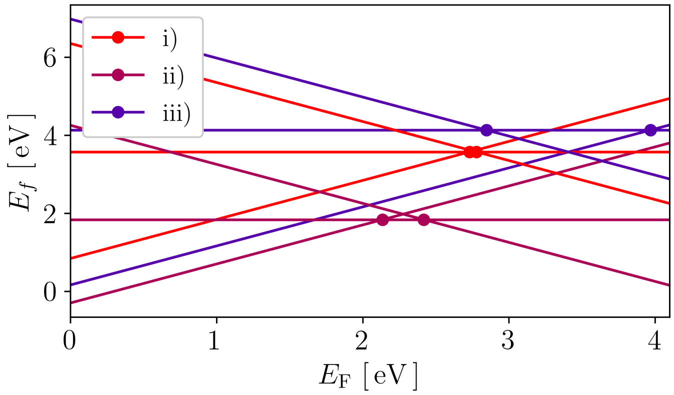

b)

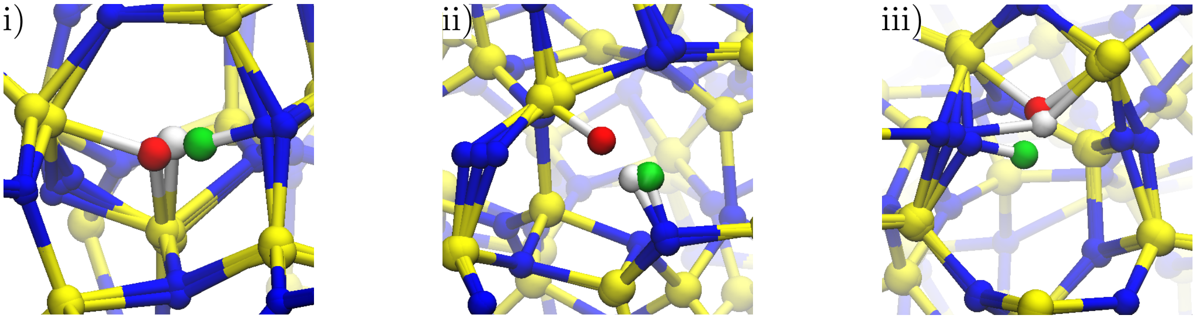

Figure S15: a) The +1/0 and 0/ − 1 charge transition levels (CTL) of the special cases marked in Figure 3f of the main article by black circles, which have a notably different electronic structure causing H-induced trapping. b) Illustration of the charge-dependent structural relaxation for these three cases. Hydrogen is colored according to the charge state of the system in green ($q = +1$), white ($q = 0$), and red ($q = -1$), Si is colored blue and N is yellow.

References

(S1) Zhang, S. B.; Northrup, J. E. Chemical potential dependence of defect formation energies in GaAs: Application to Ga self-diffusion. *Phys. Rev. Lett.* **1991**, *67*, 2339–2342, DOI: 10.1103/PhysRevLett.67.2339.

(S2) Lany, S.; Zunger, A. Accurate prediction of defect properties in density functional supercell calculations. *Model. Simul. Mat. Sci. Eng.* **2009**, *17*, 084002, DOI: 10.1088/0965-0393/17/8/084002.

(S3) Adabifiroozjaei, E.; Mofarah, S. S.; Ma, H.; Jiang, Y.; Assadi, M. H. N.; Suzuki, T. S. Molecular dynamics simulation of vacancy cluster formation in β- and α-Si3N4. *Comput. Mater. Sci.* **2020**, *178*, 109632, DOI: 10.1016/j.commatsci.2020.109632.

(S4) Le, V. V.; Dinh, T. H. Tensile deformation mechanism of amorphous silicon nitride: Insights from molecular dynamics simulations. *J. Non-Cryst. Solids* **2022**, *581*, 121381, DOI: 10.1016/j.jnoncrysol.2021.121381.

(S5) Marian, C. M.; Gastreich, M.; Gale, J. D. Empirical two-body potential for solid silicon nitride, boron nitride, and borosilazane modifications. *Phys. Rev. B* **2000**, *62*, 3117–3124, DOI: 10.1103/PhysRevB.62.3117.

(S6) Nosé, S. A unified formulation of the constant temperature molecular dynamics methods. *J. Chem. Phys.* **1984**, *81*, 511–519, DOI: 10.1063/1.447334.

(S7) Nosé, S. A molecular dynamics method for simulations in the canonical ensemble. *Mol. Phys.* **1984**, *52*, 255–268, DOI: 10.1080/00268978400101201.

(S8) Hoover, W. G. Canonical dynamics: Equilibrium phase-space distributions. *Phys. Rev. A* **1985**, *31*, 1695–1697, DOI: 10.1103/physreva.31.1695.

(S9) Thompson, A. P.; Aktulga, H. M.; Berger, R.; Bolintineanu, D. S.; Brown, W. M.; Crozier, P. S.; in 't Veld, P. J.; Kohlmeyer, A.; Moore, S. G.; Nguyen, T. D.;

Shan, R.; Stevens, M. J.; Tranchida, J.; Trott, C.; Plimpton, S. J. LAMMPS - a flexible simulation tool for particle-based materials modeling at the atomic, meso, and continuum scales. *Comput. Phys. Commun.* **2022**, *271*, 108171, DOI: 10.1016/j.cpc.2021.108171.

(S10) Hückmann, L.; Cottom, J.; Meyer, J. Intrinsic charge trapping and reversible charge induced structural modifications in a-Si_3N_4. *Adv. Phys. Res.* **2023**, 2300109, DOI: 10.1002/apxr.202300109.

(S11) Aiyama, T.; Fukunaga, T.; Niihara, K.; Hirai, T.; Suzuki, K. An X-ray diffraction study of the amorphous structure of chemically vapor-deposited silicon nitride. *J. Non-Cryst. Solids* **1979**, *33*, 131–139, DOI: 10.1016/0022-3093(79)90043-7.

(S12) Misawa, M.; Fukunaga, T.; Niihara, K.; Hirai, T.; Suzuki, K. Structure characterization of CVD amorphous Si_3N_4 by pulsed neutron total scattering. *J. Non-Cryst. Solids* **1979**, *34*, 313–321, DOI: 10.1016/0022-3093(79)90018-8.

(S13) Wakita, K. W. K.; Hayashi, H. H. H.; Nakayama, Y. N. Y. Structural study of amorphous SiN_x: H films produced by plasma-enhanced chemical vapor deposition. *Jpn. J. Appl. Phys.* **1996**, *35*, 2557, DOI: 10.1143/JJAP.35.2557.

(S14) Kang, G.; Lee, D.; Lee, K.; Kim, J.; Han, S. First-principles study on the negative-U behavior of K centers in amorphous Si_3N_4-x. *Phys. Rev. Appl.* **2018**, *10*, 064052, DOI: 10.1103/PhysRevApplied.10.064052.

(S15) Hintzsche, L.; Fang, C.; Watts, T.; Marsman, M.; Jordan, G.; Lamers, M.; Weeber, A.; Kresse, G. Density functional theory study of the structural and electronic properties of amorphous silicon nitrides: Si3N4- x: H. *Phys. Rev. B* **2012**, *86*, 235204, DOI: 10.1103/PhysRevB.86.235204.

(S16) Hintzsche, L. E.; Fang, C. M.; Marsman, M.; Jordan, G.; Lamers, M. W. P. E.; Weeber, A. W.; Kresse, G. Defects and defect healing in amorphous $Si_3N_{4-x}H_y$: An

ab initio density functional theory study. *Phys. Rev. B* **2013**, *88*, 155204, DOI: 10.1103/PhysRevB.88.155204.

(S17) Kroll, P. Structure and reactivity of amorphous silicon nitride investigated with density-functional methods. *J. Non-Cryst. Solids* **2001**, *293-295*, 238–243, DOI: 10.1016/S0022-3093(01)00676-7.

(S18) Virtanen, P.; Gommers, R.; Oliphant, T. E.; Haberland, M.; Reddy, T.; Cournapeau, D.; Burovski, E.; Peterson, P.; Weckesser, W.; Bright, J.; van der Walt, S. J.; Brett, M.; Wilson, J.; Millman, K. J.; Mayorov, N.; Nelson, A. R. J.; Jones, E.; Kern, R.; Larson, E.; Carey, C. J.; Polat, İ.; Feng, Y.; Moore, E. W.; VanderPlas, J.; Laxalde, D.; Perktold, J.; Cimrman, R.; Henriksen, I.; Quintero, E. A.; Harris, C. R.; Archibald, A. M.; Ribeiro, A. H.; Pedregosa, F.; van Mulbregt, P.; SciPy 1.0 Contributors SciPy 1.0: Fundamental algorithms for scientific computing in Python. *Nature Methods* **2020**, *17*, 261–272, DOI: 10.1038/s41592-019-0686-2.

(S19) Kühne, T. D.; Iannuzzi, M.; Del Ben, M.; Rybkin, V. V.; Seewald, P.; Stein, F.; Laino, T.; Khaliullin, R. Z.; Schütt, O.; Schiffmann, F.; Golze, D.; Wilhelm, J.; Chulkov, S.; Bani-Hashemian, M. H.; Weber, V.; Borštnik, U.; Taillefumier, M.; Jakobovits, A. S.; Lazzaro, A.; Pabst, H.; Müller, T.; Schade, R.; Guidon, M.; Andermatt, S.; Holmberg, N.; Schenter, G. K.; Hehn, A.; Bussy, A.; Belleflamme, F.; Tabacchi, G.; Glöß, A.; Lass, M.; Bethune, I.; Mundy, C. J.; Plessl, C.; Watkins, M.; VandeVondele, J.; Krack, M.; Hutter, J. CP2K: An electronic structure and molecular dynamics software package - Quickstep: Efficient and accurate electronic structure calculations. *J. Chem. Phys.* **2020**, *152*, 194103, DOI: 10.1063/5.0007045.

(S20) VandeVondele, J.; Hutter, J. Gaussian basis sets for accurate calculations on molecular systems in gas and condensed phases. *J. Chem. Phys.* **2007**, *127*, 114105, DOI: 10.1063/1.2770708.

(S21) Goedecker; Teter; Hutter Separable dual-space Gaussian pseudopotentials. *Phys. Rev. B* **1996**, *54*, 1703–1710, DOI: 10.1103/PhysRevB.54.1703.

(S22) Hartwigsen, C.; Goedecker, S.; Hutter, J. Relativistic separable dual-space Gaussian pseudopotentials from H to Rn. *Phys. Rev. B* **1998**, *58*, 3641–3662, DOI: 10.1103/PhysRevB.58.3641.

(S23) Krack, M. Pseudopotentials for H to Kr optimized for gradient-corrected exchange-correlation functionals. *Theor. Chem. Acc.* **2005**, *114*, 145–152, DOI: 10.1007/s00214-005-0655-y.

(S24) Perdew, J. P.; Burke, K.; Ernzerhof, M. Generalized gradient approximation made simple. *Phys. Rev. Lett.* **1996**, *77*, 3865–3868, DOI: 10.1103/PhysRevLett.77.3865.

(S25) Perdew, J. P.; Burke, K.; Ernzerhof, M. Erratum: "Generalized gradient approximation made simple" [Phys. Rev. Lett. 77, 3865 (1996)]. *Phys. Rev. Lett.* **1997**, *78*, 1396, DOI: 10.1103/PhysRevLett.78.1396.

(S26) Heyd, J.; Scuseria, G. E.; Ernzerhof, M. Hybrid functionals based on a screened Coulomb potential. *J. Chem. Phys.* **2003**, *118*, 8207–8215, DOI: 10.1063/1.1564060.

(S27) Heyd, J.; Scuseria, G. E.; Ernzerhof, M. Erratum: "Hybrid functionals based on a screened Coulomb potential" [J. Chem. Phys. 118, 8207 (2003)]. *J. Chem. Phys.* **2006**, *124*, 219906, DOI: 10.1063/1.2204597.

(S28) Guidon, M.; Hutter, J.; VandeVondele, J. Auxiliary density matrix methods for Hartree-Fock exchange calculations. *J. Chem. Theor. and Comp.* **2010**, *6*, 2348–2364, DOI: 10.1021/ct1002225.

(S29) Grillo, M.-E.; Elliott, S. D.; Freysoldt, C. Native defects in hexagonal β-Si_3N_4 studied using density functional theory calculations. *Phys. Rev. B* **2011**, *83*, 085208, DOI: 10.1103/PhysRevB.83.085208.

(S30) Heyd, J.; Scuseria, G. E.; Ernzerhof, M. Hybrid functionals based on a screened Coulomb potential. *J. Chem. Phys.* **2003**, *118*, 8207–8215, DOI: 10.1063/1.1564060.

(S31) Heyd, J.; Scuseria, G. E.; Ernzerhof, M. Erratum: "Hybrid functionals based on a screened Coulomb potential" [J. Chem. Phys. 118, 8207 (2003)]. *J. Chem. Phys.* **2006**, *124*, 219906, DOI: 10.1063/1.2204597.

(S32) Marian, C. M.; Gastreich, M.; Gale, J. D. Empirical two-body potential for solid silicon nitride, boron nitride, and borosilazane modifications. *Phys. Rev. B* **2000**, *62*, 3117–3124, DOI: 10.1103/PhysRevB.62.3117.

(S33) Chu, T. L.; Lee, C. H.; Gruber, G. A. The preparation and properties of amorphous silicon nitride films. *J. Electrochem. Soc.* **1967**, *114*, 717–722, DOI: 10.1149/1.2426715.

(S34) Khan, A.; Philip, J.; Hess, P. Young's modulus of silicon nitride used in scanning force microscope cantilevers. *J. Appl. Phys.* **2004**, *95*, 1667–1672, DOI: 10.1063/1.1638886.

(S35) Resende, J.; Fuard, D.; Le Cunff, D.; Tortai, J.-H.; Pelissier, B. Hybridization of ellipsometry and energy loss spectra from XPS for bandgap and optical constants determination in SiON thin films. *Mater. Chem. Phys.* **2021**, *259*, 124000, DOI: 10.1016/j.matchemphys.2020.124000.

(S36) Gritsenko, V. A.; Perevalov, T. V.; Orlov, O. M.; Krasnikov, G. Y. Nature of traps responsible for the memory effect in silicon nitride. *Appl. Phys. Lett.* **2016**, *109*, DOI: 10.1063/1.4959830.

(S37) Deák, P.; Frauenheim, T.; Gali, A. Limits of the scaled shift correction to levels of interstitial defects in semiconductors. *Phys. Rev. B* **2007**, *75*, 153204, DOI: 10.1103/PhysRevB.75.153204.

(S38) Lambrecht, W. R. Which electronic structure method for the study of defects: A commentary. *phys. stat. sol. (b)* **2011**, *248*, 1547–1558, DOI: 10.1002/pssb.201046327.

(S39) Deak, P. Calculating the optical properties of defects and surfaces in wide band gap materials. *Physica B* **2018**, *535*, 35–43, DOI: 10.1016/j.physb.2017.06.024.

(S40) Boyd, D. R. J. Infrared Spectrum of Trideuterosilane and the Structure of the Silane Molecule. *J. Chem. Phys.* **1955**, *23*, 922–926, DOI: 10.1063/1.1742148.

(S41) Martin, J. M. L.; Lee, T. J.; Taylor, P. R. An accurate ab initio quartic force field for ammonia. *J. Chem. Phys.* **1992**, *97*, 8361–8371, DOI: 10.1063/1.463406.
